# A novel role of kallikrein-related peptidase 8 in the pathogenesis of diabetic cardiac fibrosis

**DOI:** 10.7150/thno.48530

**Published:** 2021-02-20

**Authors:** Jian-Kui Du, Qing Yu, Yu-Jian Liu, Shu-Fang Du, Li-Yang Huang, Dan-Hong Xu, Xin Ni, Xiao-Yan Zhu

**Affiliations:** 1 National Clinical Research Center for Geriatric Disorders and National International Joint Research Center for Medical Metabolomics, Xiangya Hospital, Central South University, Changsha, Hunan, China.; 2 Department of Physiology, Navy Medical University, Shanghai, China.; 3 School of Kinesiology, Shanghai University of Sport, Shanghai, China.

**Keywords:** KLK8, endothelial-to-mesenchymal transition, cardiac fibrosis, diabetic cardiomyopathy, plakoglobin

## Abstract

**Rationale:** Among all the diabetic complications, diabetic cardiomyopathy, which is characterized by myocyte loss and myocardial fibrosis, is the leading cause of mortality and morbidity in diabetic patients. Tissue kallikrein-related peptidases (KLKs) are secreted serine proteases, that have distinct and overlapping roles in the pathogenesis of cardiovascular diseases. However, whether KLKs are involved in the development of diabetic cardiomyopathy remains unknown.The present study aimed to determine the role of a specific KLK in the initiation of endothelial-to-mesenchymal transition (EndMT) during the pathogenesis of diabetic cardiomyopathy.

**Methods and Results-**By screening gene expression profiles of KLKs, it was found that KLK8 was highly induced in the myocardium of mice with streptozotocin-induced diabetes. KLK8 deficiency attenuated diabetic cardiac fibrosis, and rescued the impaired cardiac function in diabetic mice. Small interfering RNA (siRNA)-mediated KLK8 knockdown significantly attenuated high glucose-induced endothelial damage and EndMT in human coronary artery endothelial cells (HCAECs). Diabetes-induced endothelial injury and cardiac EndMT were significantly alleviated in KLK8-deficient mice. In addition, transgenic overexpression of KLK8 led to interstitial and perivascular cardiac fibrosis, endothelial injury and EndMT in the heart. Adenovirus-mediated overexpression of KLK8 (Ad-KLK8) resulted in increases in endothelial cell damage, permeability and transforming growth factor (TGF)-β1 release in HCAECs. KLK8 overexpression also induced EndMT in HCAECs, which was alleviated by a TGF-β1-neutralizing antibody. A specificity protein-1 (Sp-1) consensus site was identified in the human KLK8 promoter and was found to mediate the high glucose-induced KLK8 expression. Mechanistically, it was identified that the vascular endothelial (VE)-cadherin/plakoglobin complex may associate with KLK8 in HCAECs. KLK8 cleaved the VE-cadherin extracellular domain, thus promoting plakoglobin nuclear translocation. Plakoglobin was required for KLK8-induced EndMT by cooperating with p53. KLK8 overexpression led to plakoglobin-dependent association of p53 with hypoxia inducible factor (HIF)-1α, which further enhanced the transactivation effect of HIF-1α on the TGF-β1 promoter. KLK8 also induced the binding of p53 with Smad3, subsequently promoting pro-EndMT reprogramming via the TGF-β1/Smad signaling pathway in HCAECs. The* in vitro* and *in vivo* findings further demonstrated that high glucose may promote plakoglobin-dependent cooperation of p53 with HIF-1α and Smad3, subsequently increasing the expression of TGF-β1 and the pro-EndMT target genes of the TGF-β1/Smad signaling pathway in a KLK8-dependent manner.

**Conclusions:** The present findings uncovered a novel pro-EndMT mechanism during the pathogenesis of diabetic cardiac fibrosis via the upregulation of KLK8, and may contribute to the development of future KLK8-based therapeutic strategies for diabetic cardiomyopathy.

## Introduction

The global incidence of diabetes mellitus has emerged as a major threat to worldwide health 1,2. Uncontrolled diabetes leads to a number of complications, including diabetic cardiomyopathy 3, nephropathy 4, and vision problems 5. Among all the diabetic complications, diabetic cardiomyopathy, which is characterized by myocyte loss and myocardial fibrosis, is the leading cause of mortality and morbidity in diabetic patients 3,6,7. Progressive cardiac fibrosis has been found in diabetic patients and in animal models of diabetes 7,8. Excessive collagen deposition in the myocardium reduces cardiac compliance, thus contributing to increased left ventricular stiffness and impaired contractile function, and ultimately heart failure 7,8. Despite its clinical significance, the pathological basis of diabetes-associated cardiac fibrosis remains largely unclear.

Myofibroblasts are major contributors to extracellular matrix (ECM) accumulation in fibrotic disease, and are known to be derived from resident fibroblasts, epithelial cells, and bone marrow-derived cells 9. As the initial targets of hyperglycemic damage, endothelial cells play a major role in the production of ECM proteins in all chronic diabetic complications 10. A result of sustained endothelial injury during diabetes mellitus is that endothelial cells undergo a process of transdifferentiation of endothelial cells into mesenchymal cells called endothelial-to-mesenchymal-transition (EndMT), which further switches their phenotype to myofibroblasts 11,12. Of note, EndMT is considered to be an important mechanism of diabetic cardiac fibrosis 13,14. However, the factors promoting EndMT and cardiac fibrosis during the development of diabetic cardiomyopathy require to be further elucidated.

The tissue kallikrein-related peptidase (KLK) family is a group of secreted serine proteases encoded by tandemly arranged genes of multigene families, which comprises 15 genes in human 15. The implication of KLKs in the development of fibrotic diseases and stabilization of endothelial cells has attracted considerable attention, since KLK family members are deeply involved in the degradation of ECM proteins such as fibronectin and laminin 16,17. Among KLKs, KLK1 is the member most widely studied, and confers protection against endothelial dysfunction and cardiac fibrosis induced by diabetes 18. However, the opposite effects are observed with other KLKs family members. For example, overexpression of KLK8 can eventually lead to cardiac hypertrophy and fibrosis 19. Notably, a combination of serine proteases including KLKs 1, 5 and 6, and elastases 1 and 2, can induce EndMT in human aortic endothelial cells 20. These studies indicate that KLK family members have distinct and overlapping roles in the pathogenesis of cardiovascular diseases.

By screening the mRNA expression of the KLK family in the myocardium, it was found that KLK8 was the highest induced KLK member in diabetic myocardium. Using both KLK8 knockout mice and KLK8 transgenic rats, the present study investigated the precise role of KLK8 in mediating diabetic cardiomyopathy and demonstrated that upregulation of KLK8 contributes to the development of EndMT and diabetic cardiac fibrosis. In addition, the molecular mechanisms underlying KLK8-mediated EndMT and cardiac fibrosis in the context of diabetes were illustrated.

## Results

### KLK8 expression is significantly increased in diabetic myocardium

**Figure 1:**
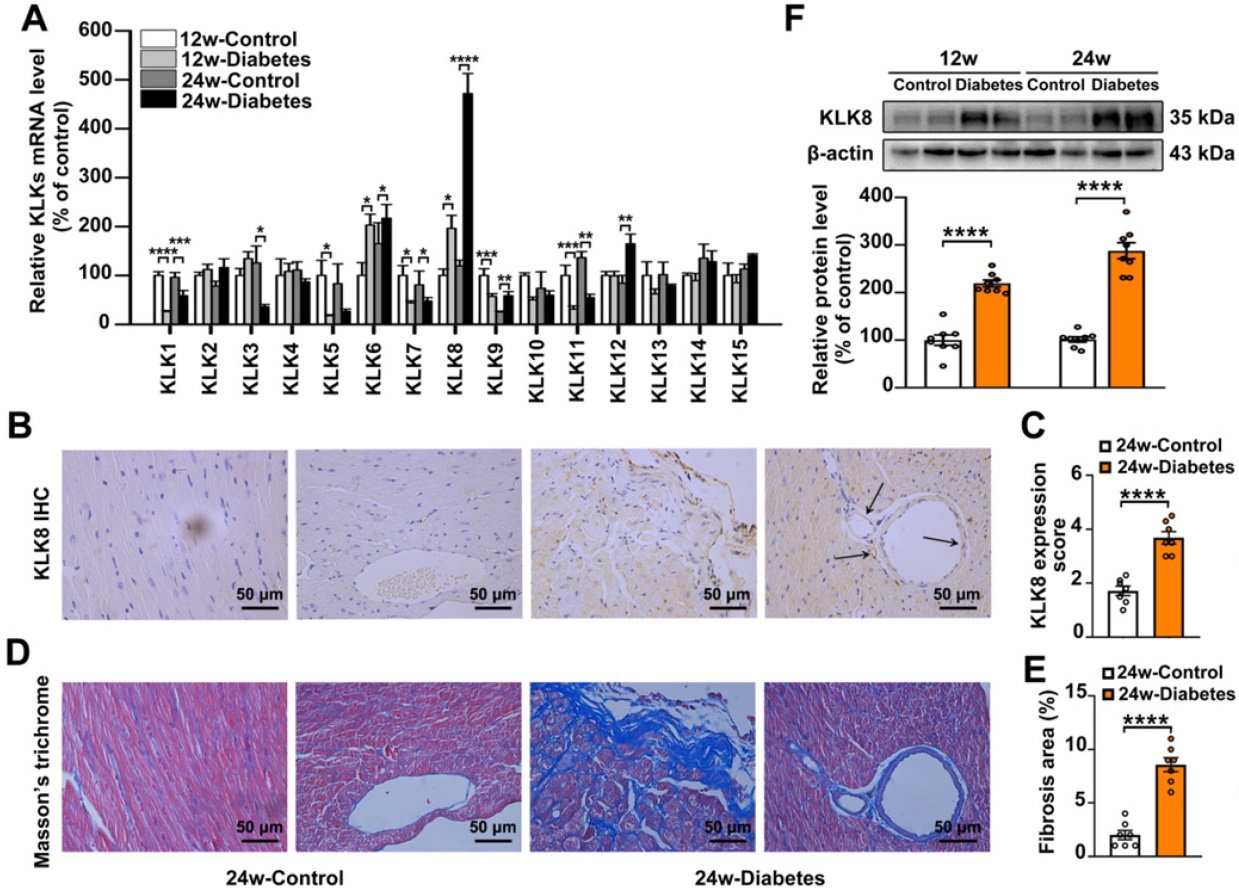
** KLK (kallikrein-related peptidase) 8 expression is significantly increased in diabetic myocardium. A,** The mRNA levels of KLK family members in the hearts after 12 and 24 weeks of diabetes. **B-E,** Immunohistochemistry (IHC) staining (B) and quantification (C) showed increased level of KLK8 in both cardiomyocytes and endothelial cells and masson's trichrome staining (D) and quantification (E) showed increased collagen deposition in both interstitial and perivascular regions in the hearts after 24 weeks of diabetes as compared to control group (scale bar = 50 µm). **F,** Immunoblots of KLK8 in the hearts after 12 and 24 weeks of diabetes. Data are expressed as means ± SEM (n = 8). *p < 0.05, **p < 0.01, ***p < 0.001, ****p < 0.0001.

To investigate the role of KLKs in the development of diabetes-associated cardiomyopathy, the present study firstly examined the mRNA expression levels of KLKs in heart tissues obtained from streptozotocin (STZ)-induced diabetic mice at 12 and 24 weeks after the induction of diabetes mellitus. As shown in Figure 1A, KLK6, KLK8 and KLK12 were significantly upregulated, while KLK1, KLK5, KLK7 and KLK11 were downregulated in the heart tissues of STZ-induced diabetic mice compared with the levels exhibited by age-matched non-diabetic controls. Among all the upregulated KLK family members, KLK8 was the highest induced KLK.

Expression of KLK8 in diabetic myocardium was then determined by immunohistochemistry staining and western blotting. As shown in Figure 1B-C, KLK8 staining was significantly increased in both cardiomyocytes and coronary endothelial cells in the myocardium of diabetic mice compared with the expression levels exhibited by the control group. As expected, Masson's trichrome staining revealed a significant collagen deposition in both interstitial and perivascular regions in the diabetic myocardium (Figure 1D-E). Immunoblotting also confirmed the induction of KLK8 protein expression in the myocardium of diabetic mice (Figure 1F).

### KLK8 deficiency attenuates diabetic cardiac fibrosis

**Figure 2:**
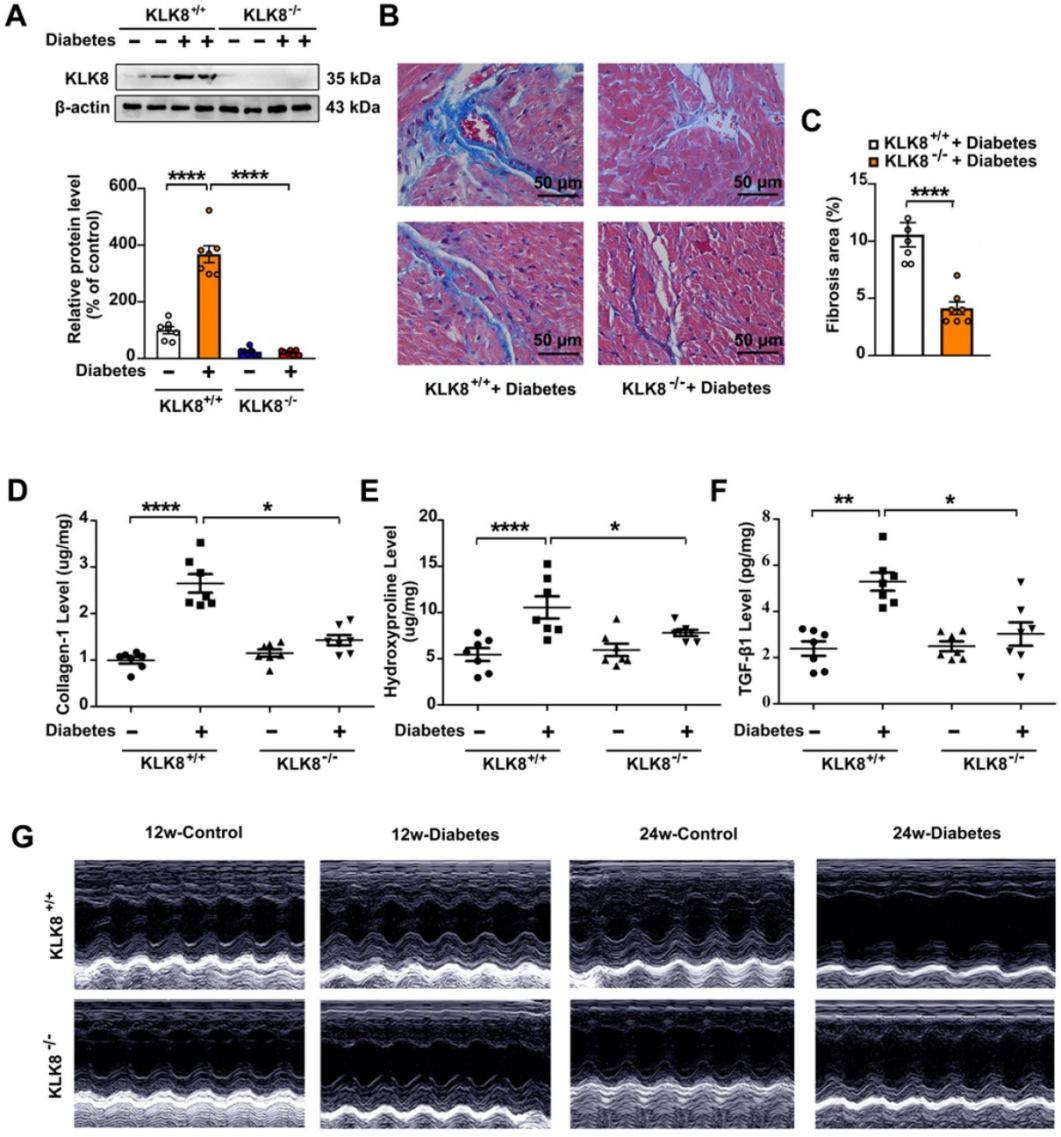
** KLK (kallikrein-related peptidase) 8 deficiency attenuates diabetic cardiac fibrosis. A-E,** Heart tissues were obtained from the KLK8-deficient (KLK8^-/-^) and KLK8^+/+^ mice after 24 weeks of diabetes. A, Immunoblots of KLK8 in the hearts. B and C, Masson's trichrome staining (B) and quantification (C) showed decreased collagen deposition in both interstitial and perivascular regions in the hearts obtained from KLK8^-/-^ diabetic mice as compared to KLK8^+/+^ diabetic mice (scale bar = 50 µm). **D-F,** Cardiac fibrosis was also quantified by determination of collagen-I (D), hydroxyproline (E), and TGF-β1 (F) levels. Data are expressed as means ± SEM (n = 7). *p < 0.05,.**p < 0.01, ***p < 0.001, ****p < 0.0001. **G,** Representative M-mode images of echocardiographic analysis exhibited smaller left ventricular end-diastolic and end-systolic dimension in KLK8^-/-^ diabetic mice as compared to KLK8^+/+^ diabetic mice after 12 and 24 weeks of diabetes.

Mice with global deletion of KLK8 were then used to investigate whether KLK8 deficiency affects diabetes-associated cardiac fibrosis. As shown in Figure 2A, the diabetes-induced upregulation of cardiac KLK8 was blunt in KLK8-deficient (KLK8^-/-^) mice. STZ-induced low insulin levels and hyperglycemia occurred in both KLK8^-/-^ and KLK8^+/+^ mice, whereas the levels of insulin and blood glucose in non-diabetic mice were normal (Table 1, Figure S1). Under baseline conditions, KLK8^-/-^ mice exhibited similar levels of body weight, total cholesterol (TC), triglyceride (TG), free fatty acid (FFA), low density lipoprotein-cholesterol (LDL-C) and high density lipoprotein-cholesterol (HDL-C) to those found in their KLK8^+/+^ littermates (Figure S1). STZ-induced diabetic mice exhibited increased levels of TC, TG, FFA and LDL-C, as well as decreased body weight and HDL-C levels. The plasma levels of TC and FFA were significantly lower in KLK8^-/-^ diabetic mice compared with those in KLK8^+/+^ diabetic mice. However, KLK8^-/-^ diabetic mice exhibited similar levels of body weight, TG, LDL-C and HDL-C to those observed in KLK8^+/+^ diabetic mice. Under baseline conditions, KLK8^-/-^ mice exhibited similar blood pressure as their KLK8^+/+^ littermates. Hyperglycemia did not increase blood pressure in the early stage (12 weeks) of the disease. However, 24 weeks of diabetes mellitus led to significant increases in systolic, diastolic and mean arterial blood pressure in KLK8^+/+^ mice, which were significantly attenuated in KLK8^-/-^ mice (Table 1).

As shown in Figure 2, KLK8^-/-^ diabetic mice displayed less blue-stained collagen deposition in both interstitial and perivascular regions (Figure 2B-C) compared with that of KLK8^+/+^ diabetic mice. In agreement with the histological observations, the concentrations of collagen I and hydroxyproline in the myocardium were significantly lower in KLK8^-/-^ diabetic mice compared with those of KLK8^+/+^ diabetic mice (Figure 2D-E). In addition, KLK8^-/-^ mice displayed a significant reduction in TGF-β1 level in the myocardium (Figure 2F).

**Figure 3:**
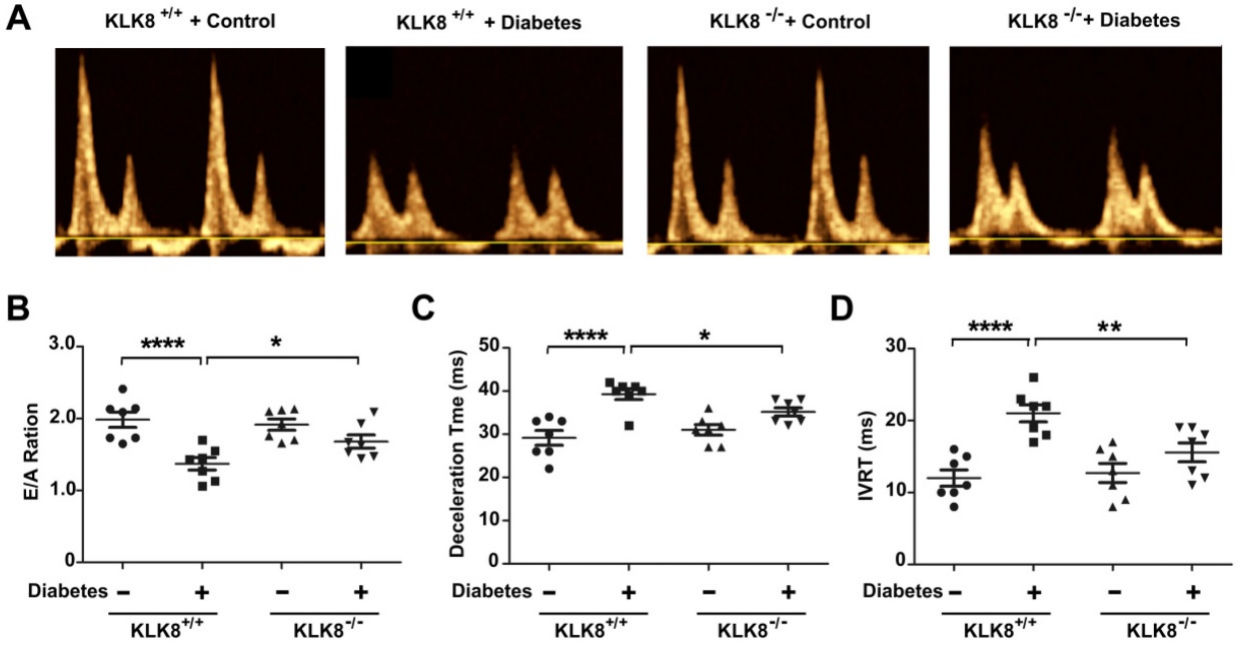
** KLK (kallikrein-related peptidase) 8 deficiency attenuates diabetic cardiac diastolic dysfunction.** Both wild-type (KLK8^+/+^) and KLK8-deficient (KLK8^-/-^) mice were injected with STZ to construct diabetic mice. Cardiac diastolic function was assessed by using echocardiographic analysis at 16 weeks after STZ injection. **A,** Representative pulse doppler images of echocardiographic analysis. **B-D,** KLK8 deficiency partially rescued the reduced E/A ratio (B), and reduced E-wave deceleration time (C) and IVRT (D) in STZ-induced diabetic mice. E/A, ratio of E wave to A wave amplitude; Deceleration Time, E-wave deceleration time; IVRT, isovolumic relaxation time. Data are expressed as means ± SEM (n = 7). *p < 0.05, **p < 0.01, ****p < 0.0001.

The present study next assessed cardiac function by using echocardiographic analysis, and observed an increase in left ventricular end-diastolic and end-systolic dimensions and a decrease in systolic function at 24 weeks after the induction of diabetes mellitus (Figure 2G, Table 2). KLK8^-/-^ mice exhibited significantly smaller left ventricular end-diastolic and end-systolic dimension and higher fractional shortening and ejection fraction than KLK8^+/+^ mice (Figure 2G, Table 2). Cardiac fibrosis is closely associated with cardiac diastolic dysfunction 21, 22. The present study next observed the effect of KLK8 deficiency on diabetes-induced diastolic dysfunction. As shown in Figure 3, the E/A ratio was significantly decreased, whereas the E-wave deceleration time and isovolumic relaxation time (IVRT) were markedly increased in STZ-induced diabetic mice at 16 weeks after the induction of diabetes mellitus, indicating the occurrence of diastolic dysfunction in diabetic mice. It was found that KLK8 deficiency partially rescued the reduced E/A ratio, and reduced E-wave deceleration time and IVRT in STZ-induced diabetic mice, suggesting that KLK8 deficiency prevented further deterioration of diastolic function during diabetes progression. These findings indicate that KLK8 upregulation contributes to cardiac fibrosis and impaired cardiac function in diabetic mice.

### Upregulation of KLK8 contributes to high glucose-induced endothelial damage and EndMT

**Figure 4:**
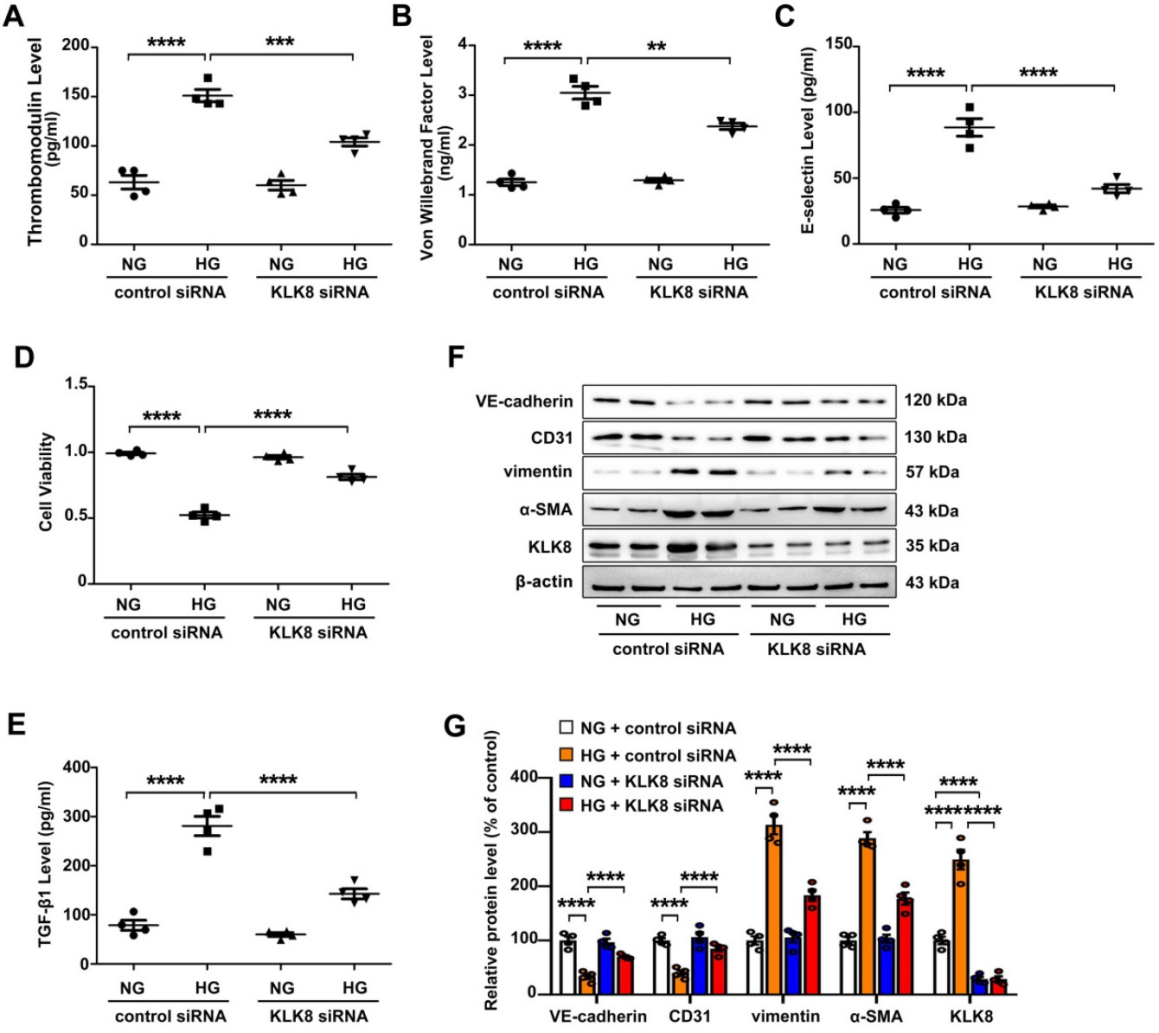
** KLK (kallikrein-related peptidase) 8 knockdown alleviates high glucose-induced endothelial dysfunction and endothelial-to-mesenchymal transition in human coronary artery endothelial cells (HCAECs).** HCAECs were transfected with control siRNA or KLK8 siRNAs, and were cultured with or without high glucose (HG, 25 mM) treatment for 5 days. **A-C,** Levels of thrombomodulin (A), von Willebrand factor (B) and E-selectin (C) as markers of endothelial damage/dysfunction and activation in the culture medium. **D,** MTT assay showed that KLK8 knockdown alleviates HG-induced cell injury in HCAECs. **E,** KLK8 knockdown alleviates HG-induced TGF-β1 release in the supernatant of HCAECs. **F & G,** Immunoblots of the endothelial markers (VE-cadherin, CD31) and mesenchymal markers (vimentin, α-SMA). The representative protein bands (F) and the corresponding histograms (G) showed that HG-induced loss of CD31 and VE-cadherin was largely prevented, whereas the acquisition of α-SMA and vimentin was decreased by KLK8 knockdown. Data are expressed as means ± SEM (n = 4). **p < 0.01, ***p < 0.001, ****p < 0.0001.

High glucose is known to induce endothelial dysfunction and EndMT in cardiac endothelial cells 13,14. It was found that high glucose (25 mM) treatment for 5 days caused endothelial cell damage, as revealed by increased release of endothelial damage markers 23 including thrombomodulin, von Willebrand factor (VWF) and E-selectin (Figure 4A-C). High glucose treatment also led to significant decrease in cell viability (Figure 4D). In addition, TGF-β1 release was elevated in high glucose-treated human coronary artery endothelial cells (HCAECs) (Figure 4E). High glucose treatment also increased the expression levels of α-SMA and vimentin, whereas it decreased the expression levels of CD31 and VE-cadherin in HCAECs (Figure 4F-G). These results confirm that high glucose alone is able to induce endothelial damage and EndMT in HCAECs.

The present study next explored whether upregulation of KLK8 contributes to high glucose-induced endothelial dysfunction and EndMT using an *in vitro* model. KLK8 siRNA not only led to a significant decrease of KLK8 expression in HCAECs, but also blocked the high glucose-induced upregulation of KLK8. KLK8 knockdown significantly attenuated high glucose-induced endothelial damage (Figure 4A-D). Furthermore, the high glucose-induced increase in TGF-β1 release and EndMT was largely prevented by KLK8 knockdown in HCAECs (Figure 4E-G).

**Figure 5:**
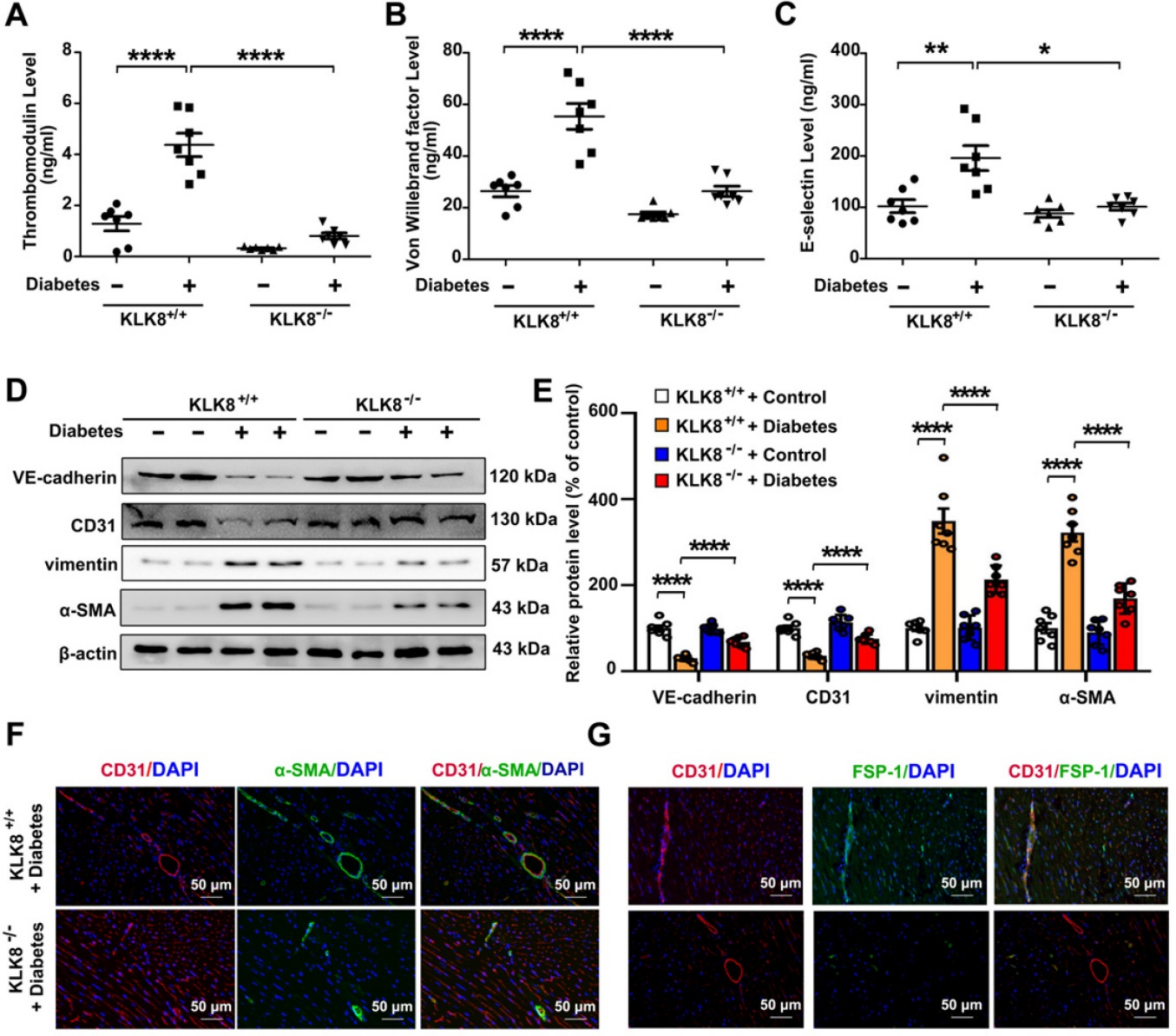
** KLK (kallikrein-related peptidase) 8 deficiency attenuates diabetic endothelial damage and endothelial-to-mesenchymal transition.** Plasma samples and heart tissues were obtained from the KLK8-deficient (KLK8^-/-^) and KLK8^+/+^ mice after 24 weeks of diabetes. **A-C,** Plasma levels of thrombomodulin (A), von Willebrand factor (B) and E-selectin (C). **D & E,** Immunoblots of VE-cadherin, CD31, vimentin and α-SMA in the hearts. The representative protein bands (D) and the corresponding histograms (E) showed that the loss of CD31 and VE-cadherin in diabetic heart tissues was largely prevented, whereas the acquisition of α-SMA and vimentin was dramatically decreased in KLK^-/-^ mice. **F & G,** Immunofluorescent staining showed decreased colocalization of α-SMA (F, green), FSP-1 (G, green) and CD31 (red) in the hearts obtained from KLK8^-/-^ diabetic mice as compared to KLK8^+/+^ diabetic mice. Nuclei were counterstained with DAPI (blue), scale bar = 50 µm. Data are expressed as means ± SEM (n = 7). *p < 0.05, **p < 0.01, ***p < 0.001, ****p < 0.0001.

In the animal model, diabetes mellitus led to an increase in the plasma levels of thrombomodulin, VWF and E-selectin in KLK8^+/+^ mice, which were significantly attenuated in KLK8^-/-^ mice (Figure 5A-C). It was found that the loss of CD31 and VE-cadherin in diabetic heart tissues was largely prevented, whereas the expression of α-SMA and vimentin was markedly decreased in KLK8^-/-^ mice (Figure 5D-E). Double immunofluorescence staining was then performed with antibodies against CD31, α-SMA, FSP-1 and vimentin. It was found that KLK8^-/-^ mice exhibited less colocalization of CD31/α-SMA (Figure 5F), CD31/FSP-1 and CD31/vimentin (Figure 5G, Figure S2) in heart tissues compared with the findings in KLK^+/+^ mice.

### KLK8 overexpression leads to interstitial and perivascular cardiac fibrosis, endothelial damage and EndMT

**Figure 6:**
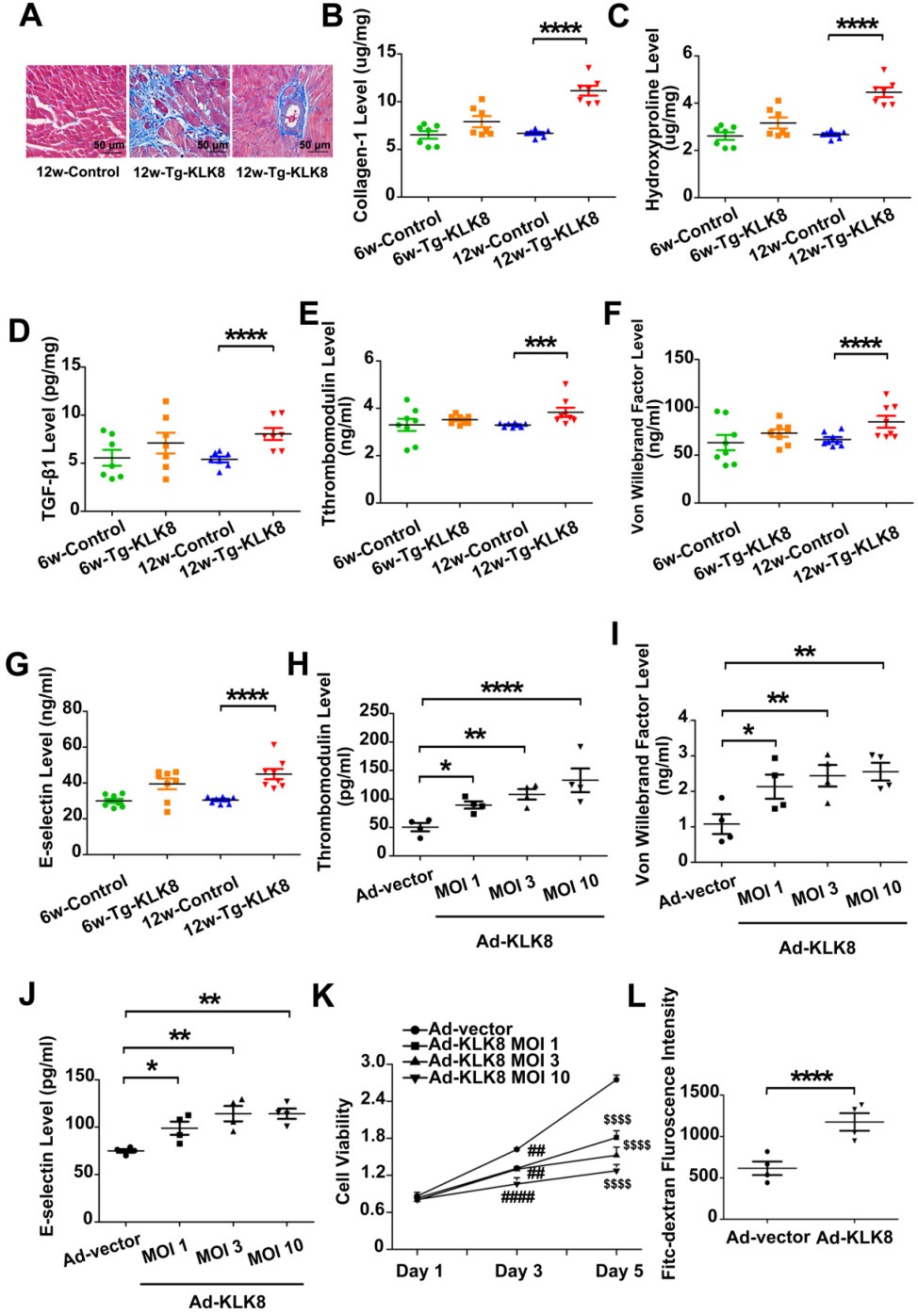
** KLK (kallikrein-related peptidase) 8 overexpression leads to cardiac fibrosis and endothelial damage. A,** Masson's trichrome staining showed collagen deposition in the perivascular and intramyocardial regions in the hearts obtained from 12-weeks-old Tg-KLK8 rats (scale bar = 50 µm). **B-D,** Cardiac fibrosis was also quantified by determination of collagen-I (B), hydroxyproline (C), and TGF-β (D) levels in heart tissues obtained from 6-weeks-old and 12-weeks-old control and Tg-KLK8 rats. **E-G,** Plasma levels of thrombomodulin (E), von Willebrand factor (F) and E-selectin (G) as markers of endothelial damage/dysfunction and activation in 6-weeks-old and 12-weeks-old control and KLK8 transgenic (Tg-KLK8) rats. Data are expressed as means ± SEM (n = 7). ***p < 0.001, ****p < 0.0001. H-K, HCAECs were infected with increasing doses of KLK8 adenovirus (Ad-KLK8) at a multiplicity of infection (MOI) of 1, 3, or 10 for 72 h. **H-J,** Levels of thrombomodulin (H), von Willebrand factor (I) and E-selectin (J) in the culture medium. **K,** MTT assay showed that Ad-KLK8 induced cell injury in a dose- and time-dependent manner in human coronary artery endothelial cells (HCAECs). **L,** The permeability of a confluent HCAECs monolayer measured by FITC-dextran flux assay showed that Ad-KLK8 (MOI 10) treatment for 72 h led to significantly increased permeability of HCAECs. Data are expressed as means ± SEM (n = 4). *p < 0.05, **p < 0.01, ****p < 0.0001, ##*p* < 0.01, ####*p* < 0.0001 versus Ad-vector Day 3; $$$$*p* < 0.0001 versus Ad-vector Day 5.

Using KLK8 transgenic rats 19, the present study examined the characteristics of cardiac fibrosis caused by KLK8 overexpression. Compared with those of age-matched control rats, 6-weeks-old and 12-weeks-old KLK8 transgenic rats had similar body weights (Figure S3). Masson's trichrome staining demonstrated prominent collagen deposition in the interstitial and perivascular regions of the myocardium obtained from 12-weeks-old KLK8 transgenic rats (Figure 6A). As expected, the concentration of collagen, hydroxyproline and TGF-β1 was significantly elevated in heart tissues of 12-weeks-old KLK8 transgenic rats as compared with that of control rats (Figure 6B-D). The present study then investigated whether KLK8 overexpression leads to endothelial injury, and found that the circulatory levels of thrombomodulin, VWF and E-selectin, as markers of endothelial damage and activation 23, were significantly increased in 12-weeks-old transgenic rats compared with those of age-matched control rats (Figure 6E-G).

The present study further confirmed the effect of KLK8 on endothelial cells. Infection of HCAECs with increasing concentrations of KLK8 adenovirus (Ad-KLK8) led to an increase in KLK8 expression in a dose-dependent manner (Figure S4). Furthermore, endothelial cell damage occurred, as revealed by an increase in the release of thrombomodulin, VWF and E-selectin, as well as a decrease in cell viability (Figure 6H-K). In addition, the permeability of a confluent HCAEC monolayer was measured for 10-kDa FITC-dextran, and it was found that Ad-KLK8 treatment significantly increased endothelial permeability (Figure 6L).

Cardiac fibroblasts are well known to play critical roles in the pathogenesis of diabetic cardiac fibrosis 24, 25. The effect of KLK8 on cardiac fibroblasts was next evaluated. As shown in supplemental Figure S5, treatment of cardiac fibroblasts with Ad-KLK8 induced mRNA expression of the proliferation-related genes Ki67, proliferating cell nuclear antigen (PCNA) and cyclin D1 26. Protein expression levels of Ki67 and PCNA were also increased by KLK8 overexpression. Cell proliferation assay and Ki67 immunostaining revealed that KLK8 overexpression markedly enhanced the proliferation of cardiac fibroblasts. Additionally, transwell migration assays were performed, and it was found that KLK8 overexpression significantly increased migration in cardiac fibroblasts. These *in vitro* data indicated that KLK8 selectively injured cardiac endothelial cells, whereas it promoted proliferation and migration in cardiac fibroblasts.

**Figure 7:**
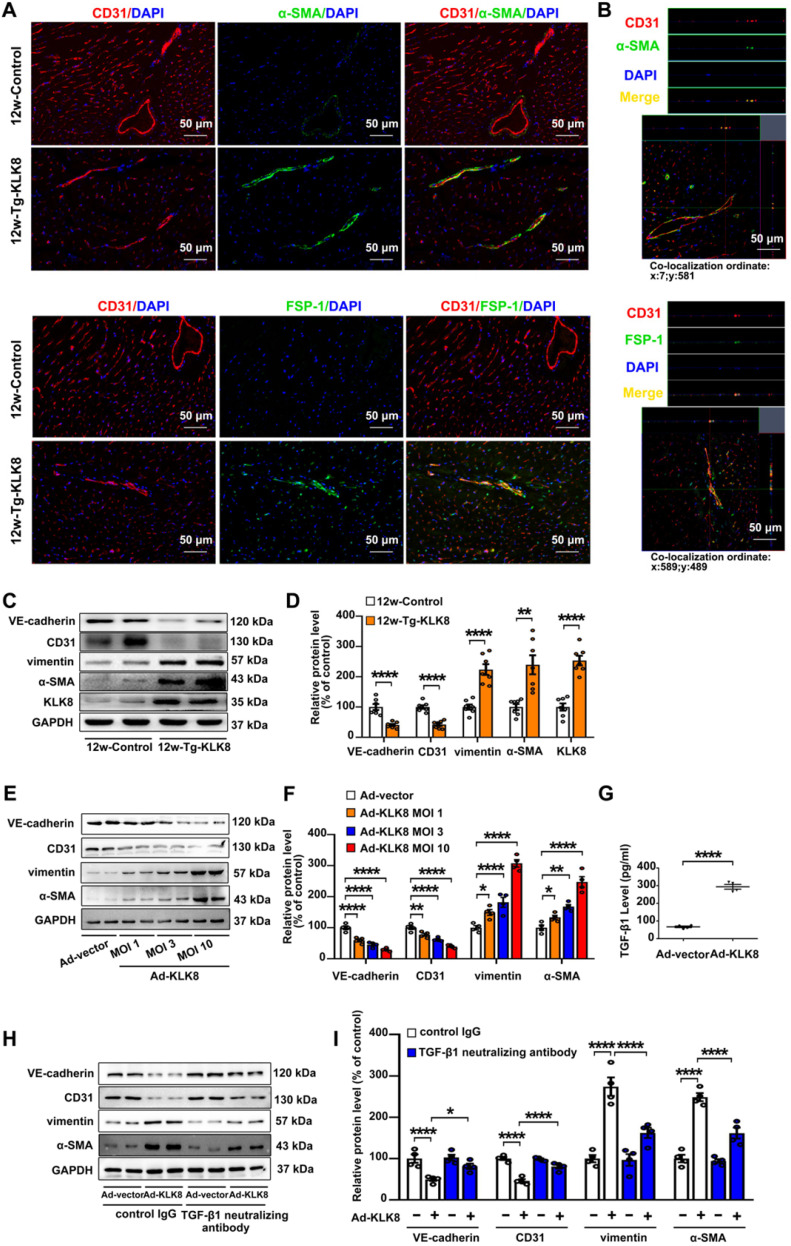
** KLK (kallikrein-related peptidase) 8 overexpression induces endothelial-to-mesenchymal transition in the myocardium and human coronary artery endothelial cells (HCAECs).** A, Immunofluorescent staining showed increased colocalization of α-SMA (green) and FSP-1 (green) and CD31 (red) in the hearts obtained from 12-weeks-old Tg-KLK8 rats as compared to age-matched control rats. Nuclei were counterstained with DAPI (blue), scale bar = 50 µm. B, Representative z-stack image analysis showed specific overlay of double immunostaining. CD31^+^/α-SMA^+^ and CD31^+^/FSP-1^+^ cells in specific ordinate were analyzed in z-stack with optimal interval range of 0.8 µm. Nuclei were counterstained with DAPI (blue), scale bar = 50 µm. C & D, Immunoblots of the VE-cadherin, CD31, vimentin, and α-SMA in the hearts obtained from 12-weeks-old control and Tg-KLK8 rats. The representative protein bands (C) and the corresponding histograms (D) showed that transgenic KLK8 overexpression significantly upregulated vimentin and α-SMA expression, whereas downregulated VE-cadherin and CD31 expression in heart tissues. E & F, Immunoblots of the endothelial markers (VE-cadherin, CD31) and mesenchymal markers (vimentin, α-SMA) in HCAECs infected with increasing doses of KLK8 adenovirus (Ad-KLK8) at a multiplicity of infection (MOI) of 1, 3, or 10 for 72 h. The representative protein bands (E) and the corresponding histograms (F) showed that Ad-KLK8 dose-dependently increased protein expressions of the mesenchymal markers, whereas decreased the endothelial markers in HCAECs. G, Infection of Ad-KLK8 at an MOI of 10 for 72 h led to an increase of TGF-β1 release in the supernatant of HCAECs. H & I, Immunoblots of the endothelial markers (VE-cadherin, CD31) and mesenchymal markers (vimentin, α-SMA). The representative protein bands (H) and the corresponding histograms (I) showed that the KLK8-induced loss of CD31 and VE-cadherin was largely prevented, whereas the acquisition of α-SMA and vimentin was decreased by TGF-β1 neutralizing antibody. Data are expressed as means ± SEM (n = 4). *p < 0.05, **p < 0.01, ****p < 0.0001.

As endothelial injury is an important initiator of EndMT, the present study next investigated the colocalization of endothelial and mesenchymal markers in the hearts of KLK8 transgenic rats. Figure 7A demonstrated colocalization of CD31 and the myofibroblast marker α smooth muscle actin (α-SMA) in the endothelium layer of cardiac vessels in the myocardium obtained from 12-weeks-old KLK8 transgenic rats. Quantification analysis showed that transgenic overexpression of KLK8 significantly increased the percentage of CD31^+^/α-SMA^+^ cells among total α-SMA+ cells compared with that of control rats (Figure S6). Other mesenchymal cell markers such as fibroblast-specific protein (FSP)-1 and vimentin 27, were also shown to be colocalized with CD31^+^ cells (Figure 7A, Figure S6). Double-positive staining was further examined by Z-stack analysis 28, which confirmed the specific overlay of CD31^+^/α-SMA^+^, CD31^+^/FSP-1^+^ and CD31^+^/vimentin^+^ cells (Figure 7B, Figure S6). The expression levels of endothelial and mesenchymal markers in the myocardium were also evaluated. As shown in Figure 7C-D, vimentin and α-SMA were significantly upregulated, whereas VE-cadherin and CD31 were downregulated in heart tissues of KLK8 transgenic rats compared with those of control rats. The loss of endothelial markers, accompanied by the acquisition of mesenchymal cell markers, suggests that KLK8 overexpression induces EndMT in the myocardium.

In HCAECs, it was found that Ad-KLK8 increased the expression levels of α-SMA and vimentin, whereas it decreased the expression levels of CD31 and VE-cadherin, in a dose-dependent manner, suggesting that KLK8 overexpression is able to induce EndMT (Figure 7E-F). TGF-β1 is a main inducer of EndMT 29. The present study found that TGF-β1 release was elevated in Ad-KLK8-infected HCAECs compared with that in Ad-vector-infected cells (Figure 7G). Notably, the KLK8-induced loss of CD31 and VE-cadherin was largely prevented, whereas the acquisition of α-SMA and vimentin was decreased, by the TGF-β1 neutralizing antibody (Figure 7H-I), suggesting that increased TGF-β1 production contributes to KLK8-induced EndMT. Taken together, these *in vivo* and *in vitro* results suggest that KLK8 overexpression leads to endothelial damage and EndMT, which may be a source of fibroblast accumulation in KLK8-induced cardiac fibrosis.

### Sp-1 mediates high glucose-induced upregulation of KLK8 in endothelial cells

**Figure 8:**
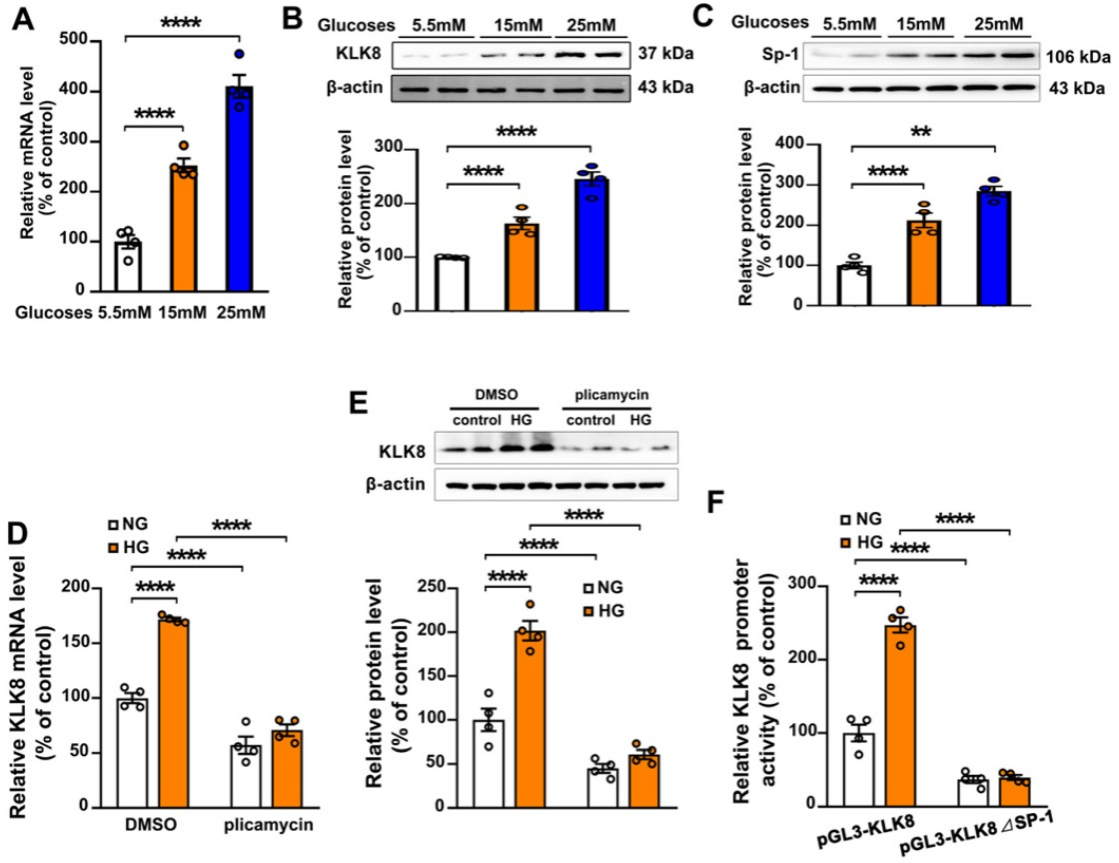
** Sp-1 mediates high glucose-induced upregulation of KLK8 in human coronary artery endothelial cells (HCAECs). A-C,** HCAECs were treated with increasing concentration of glucose (15 and 25 mM) for 5 days. A, The mRNA level of KLK8 in HCAECs. B and C, Immunoblots of KLK8 (B) and Sp-1 (C). The representative protein bands of KLK8 (B) and Sp-1 (C) were presented on the top of the corresponding histograms. **D and E,** High glucose-induced mRNA (D) and protein (E) expressions of KLK8 were abolished by Sp-1 inhibitor plicamyin (1 nM) in HCAECs. F, HCAECs were transfected with human KLK8 promoter-luciferase reporter plasmid pGL3-KLK8 or mutated plasmid pGL3-KLK8**△**Sp1, and then were exposed to high glucose (25 mM) for 48 h. Promoter activity was analyzed using a dual-luciferase reporter assay. Data are expressed as means ± SEM (n = 4). **p < 0.01, ****p < 0.0001.

To explore the molecular mechanisms involved in the high glucose-induced upregulation of KLK8 expression, HCAECs were treated with increasing concentration of glucose (15 and 25 mM) for 5 days. It was found that the mRNA and protein expression levels of KLK8 were significantly upregulated in a dose-dependent manner in high glucose-treated HCAECs compared with those of normal glucose-treated HCAECs (Figure 8A-B), suggesting that high glucose stimulated KLK8 expression at the transcriptional level. Bioinformatic analysis revealed a Sp-1 consensus sequence (5'-GGCGG-3') located within the proximal region of the KLK8 promoter. A number of studies have reported that the transcription factor Sp-1 mediates hyperglycemia-induced upregulation of various genes, thus contributing to high glucose-induced endothelial injury 30-32. Therefore, the present study explored whether high glucose-induced upregulation of KLK8 expression requires Sp-1 in HCAECs. As shown in Figure 8C, high glucose stimulated Sp-1 protein expression in a dose-dependent manner in a five-day incubation period. Sp-1 inhibitor plicamycin (1nM) totally abolished high glucose-induced up-regulation of KLK8 expression (Figure 8D-E). Furthermore, Sp-1 inhibitor also caused a decrease in basal KLK8 expression. We then generated a KLK8 report gene by cloning the promoter of human KLK8 into the pGL3 vector, thereby further confirming that high glucose stimulates KLK8 expression through the Sp-1 site in the KLK8 gene. As shown in Figure 8F, treatment of cells with high glucose (25 mM) significantly increased luciferase activity of the KLK8 reporter gene. We then generated the KLK8 reporter gene containing the mutant Sp-1 binding site and transfected the HCAEC cells with this mutated KLK8 reporter gene. The results showed that the basal luciferase activity of this mutant reporter gene was significantly decreased compared with that of the normal KLK8 reporter gene. High glucose treatment did not increase the luciferase activity of the mutant KLK8 reporter gene. These findings indicate that Sp-1 mediates high glucose-induced upregulation of KLK8 in endothelial cells.

### KLK8 degrades VE-cadherin, thereby promoting plakoglobin nuclear translocation

The KLK family is considered to cleave kininogens to release kinin 15. Kinin is well known to function through kinin B_1_ receptor (B_1_R) and B_2_R, which are present in endothelial cells 33. The present study found that Ad-KLK8 infection resulted in a 2.88 ± 0.35 fold increase in the content of bradykinin in the culture medium of HCAECs. However, knockdown of B_1_R or B_2_R could not reverse KLK8-induced endothelial cell injury or EndMT (Figure S7).

Two serine protease inhibitors, antipain and ZnSO4 were then used to block the proteolytic activity of KLK8, as described previously 19, 34. As shown in Figure S8, antipain and ZnSO4 blocked KLK8-induced endothelial cell injury and EndMT, suggesting that the pro-EndMT effect of KLK8 is dependent on its proteolytic activity.

**Figure 9:**
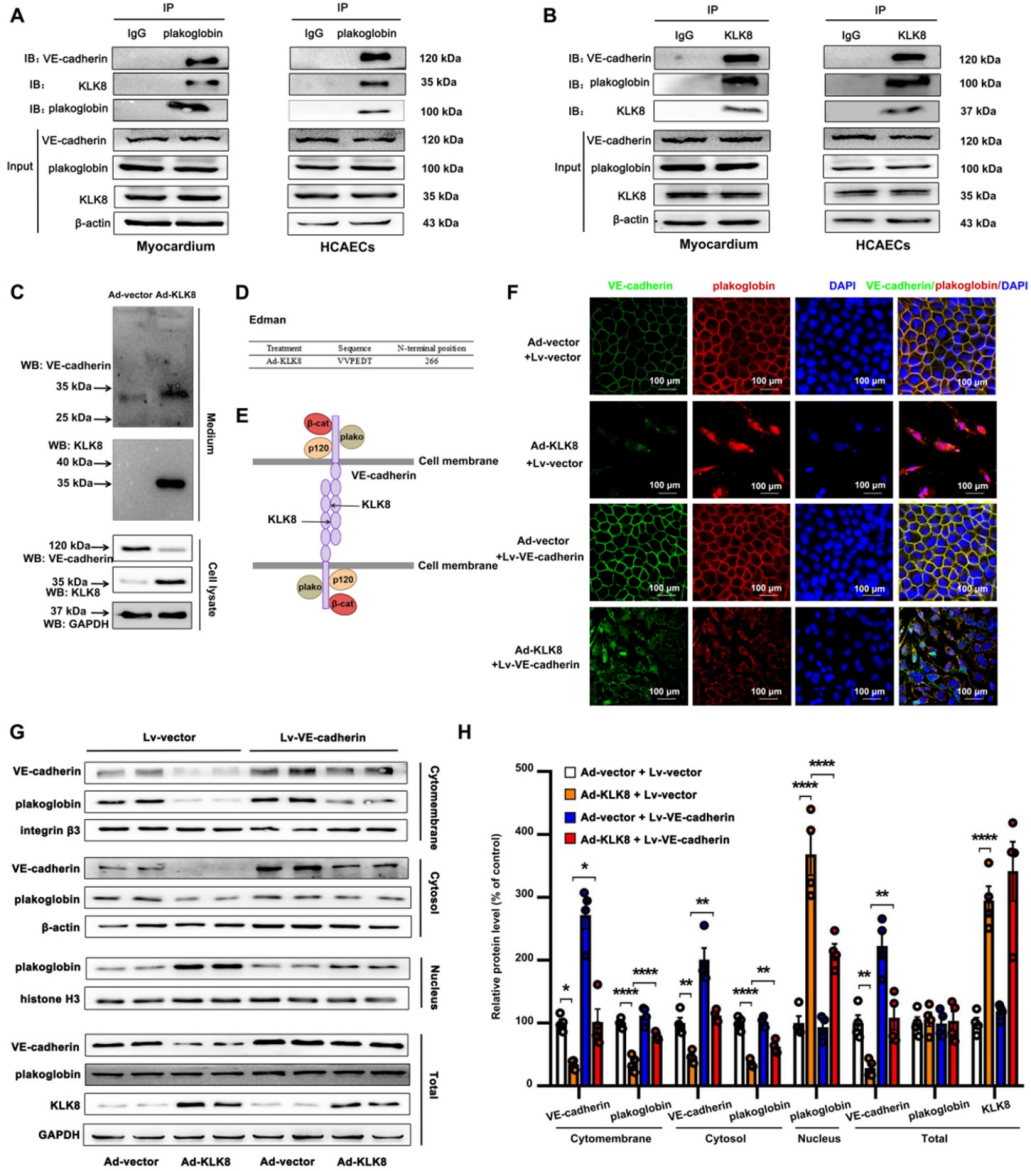
** KLK (kallikrein-related peptidase) 8 degrades VE-cadherin, thus promoting plakoglobin nuclear translocation. A and B,** Association of plakoglobin with VE-cadherin and KLK8 was tested by reciprocal immunoprecipitations in rat myocardium and human coronary artery endothelial cells (HCAECs). IgG was controlled for nonspecific interaction. **C,** HCAECs were treated with Ad-vector or Ad-KLK8 in serum-free medium for 72 h. Immunoblots showed the appearance of a ~30 kDa N-terminal VE-cadherin fragment in the medium. WB, western blot. **D,** The N-terminal sequence of the ~30 kDa protein band was determined by Edman assay. The sequence as indicated was belonging to VE-cadherin sequence. **E,** Schematic representation of the extracellular part of VE-cadherin cleaved by KLK8. VE-cadherin extracellular domain is constituted by 5 cadherin domains numbered 1-5 from the N-terminus. KLK8 cleaved VE-cadherin before amino-acid 266 in the extracellular cadherin domain 3. **F,** Immunofluorescent staining showed that infection of Ad-KLK8 for 72 h resulted in significant loss of both plakoglobin (red) and VE-cadherin (green) in the plasma membrane, whereas caused nuclear translocation of plakoglobin in HCAECs. Lentivirus-mediated VE-cadherin (Lv-VE-cadherin) overexpression reduced nuclear whereas increased membrane and cytosol plakoglobin levels. Nuclei were counterstained with DAPI (blue), scale bar = 100 µm. **G,** Immunoblots of cellular fractionations of plasma membrane, cytosol and nucleus for VE-cadherin and plakoglobin in HCAECs infected with or without Ad-KLK8 and Lv-VE-cadherin for 72 h. The representative protein bands (H) and the corresponding histograms (I) were presented. Data are expressed as means ± SEM (n = 4). **p < 0.01, ****p < 0.0001.

Using co-immunoprecipitation (Co-IP) combined with mass spectrometry, the present study identified proteins associated with KLK8 in the myocardium of KLK8 transgenic rats. Mass spectrometry revealed that plakoglobin (also called γ-catenin), a key component of adherens junctions in endothelial cells 35, was the potential matched protein (Supplementary Table S1). Plakoglobin is known to associate with the membrane indirectly via an interaction with transmembrane cell adhesion molecules of the cadherin family such as VE-cadherin 35. As shown in Figure 9A-B, plakoglobin was co-immunoprecipitated by anti-KLK8, and *vice versa,* in rat myocardium and HCAECs. Notably, VE-cadherin was also co-immunoprecipitated by anti-KLK8 (Figure 9B). KLK8 overexpression led to downregulation of VE-cadherin (Figure 9C) but had no significant effect on plakoglobin expression in myocardium or HCAECs (Figure S9). These results suggest that VE-cadherin may be recognized as a potential substrate of KLK8.

The present study then examined the composition of the endothelial cell culture medium after cell transfection with Ad-KLK8. It was found that a new ~30 kDa extracellular fragment of VE-cadherin was released into the medium (Figure 9C). N-terminal sequencing was then performed on this 30-kDa fragment, and the data showed that KLK8 cleaved VE-cadherin before amino acid 266 in the extracellular cadherin domain 3 (Figure 9D-E), suggesting the cleaved VE-cadherin protein lacks the adhesive domain of VE-cadherin.

Cleavage of VE-cadherin can lead to the release of β-catenin from the plasma membrane, and can accelerate the accumulation of β-catenin in the nucleus of endothelial cells 36,37. The present study next examined whether KLK8 affected the subcellular localization of plakoglobin. Immunofluorescence staining showed that plakoglobin was localized on the cell membrane in Ad-vector treated endothelial cells, and was mainly colocalized with VE-cadherin (Figure 9F). Notably, KLK8 overexpression resulted in a significant loss of plakoglobin and VE-cadherin in the plasma membrane, whereas it caused marked nuclear translocation of plakoglobin in HCAECs (Figure 9F). KLK8-induced nuclear translocation of plakoglobin was also confirmed by western blotting. As shown in Figure 9G-H, the membrane and cytosolic levels of plakoglobin were decreased, while the nuclear plakoglobin levels were increased, in Ad-KLK8-treated HCAECs compared with those of Ad-vector treated cells. Lentivirus-mediated VE-cadherin overexpression markedly reduced nuclear plakoglobin levels, whereas it increased membrane and cytosolic plakoglobin levels (Figure 9F-H).

### Plakoglobin is required for KLK8-induced endothelial-to-mesenchymal transition by cooperating with p53

**Figure 10:**
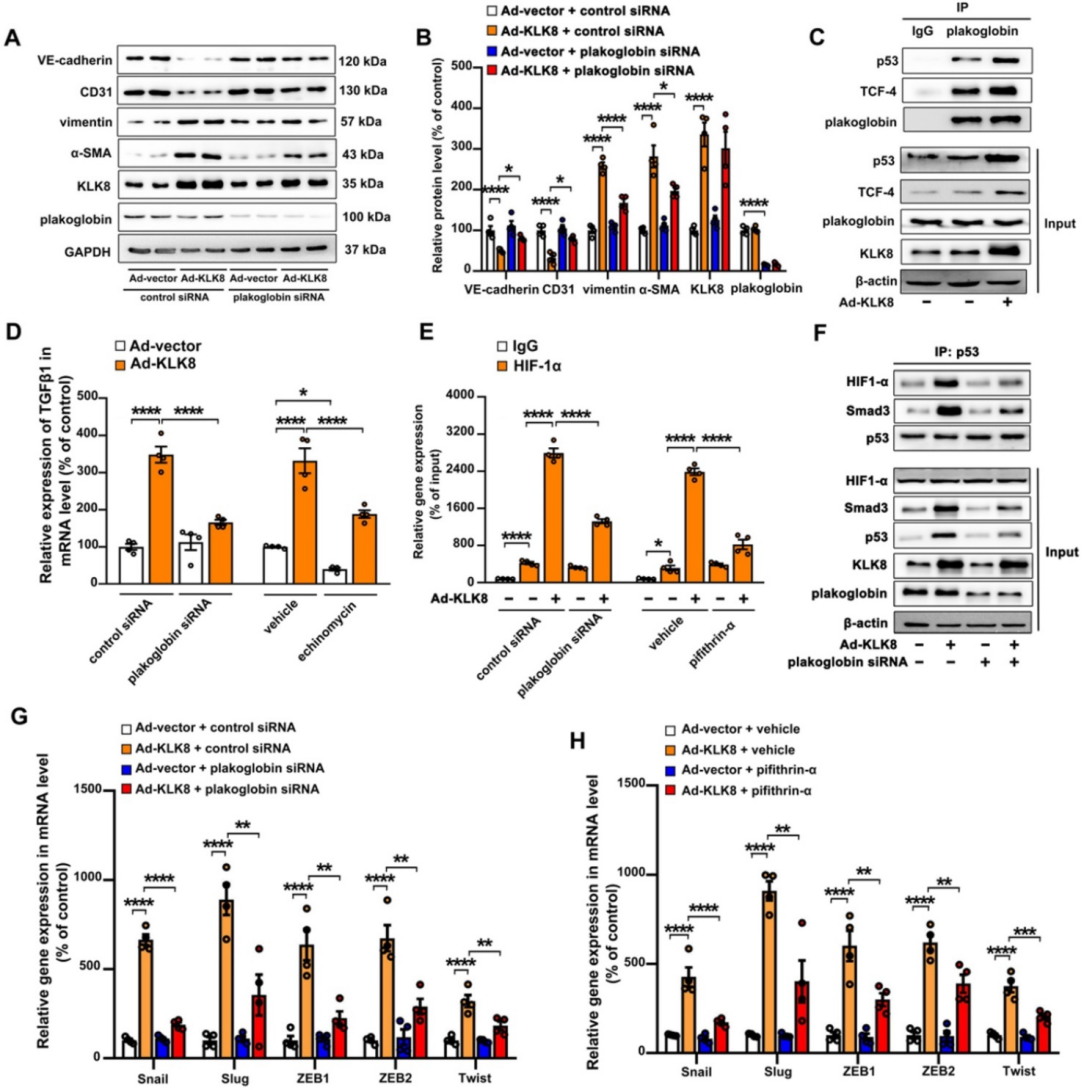
** Plakoglobin is required for KLK (kallikrein-related peptidase) 8-induced endothelial-to-mesenchymal transition by cooperating with p53. A & B,** Immunoblots of plakoglobin-knockdown human coronary artery endothelial cells (HCAECs) under KLK8 adenovirus (Ad-KLK8) treatment for 72 h. The representative protein bands (A) and the corresponding histograms (B) were presented. **C,** Association of plakoglobin with TCF-4 and p53 was observed by immunoprecipitation in HCAECs, whereas Ad-KLK8 treatment enhanced the interactions. IgG was controlled for nonspecific interaction. **D,** The KLK8 overexpression-induced mRNA expression of TGF-β1 was reduced by plakoglobin knockdown or HIF-1α inhibitor echinomycin (20 nM) in HCAECs. **E,** ChIP assay showed that the KLK8 overexpression-induced HIF-1α binding to TGF-β1 promoter was blocked by plakoglobin knockdown or p53 inhibitor pifithrin-α (20 μM). **F,** Immunoprecipitation assay showed that Ad-KLK8 treatment enhanced the association of p53 with HIF-1α and Smad3 in HCAECs, which was reduced by plakoglobin knockdown. **G and H,** The KLK8 overexpression-induced mRNA levels of the pro-EndMT target genes of TGF-β1/Smad pathway (Snail, Slug, Zeb1, Zeb2 and Twist) were reduced by plakoglobin knockdown (G) or p53 inhibitor pifithrin-α (H, 20 μM). Data are expressed as means ± SEM (n = 4). *p < 0.05, **p < 0.01, ***p < 0.001, ****p < 0.0001.

As shown in Figure 10A-B, the KLK8-induced loss of CD31 and VE-cadherin was largely prevented, whereas the upregulation of α-SMA and vimentin was markedly decreased by plakoglobin knockdown, indicating that KLK8 may promote EndMT through a plakoglobin-dependent pathway.

Plakoglobin is known to interact with transcription factors, including p53 and TCF family members 38,39, which have been implicated in the development of mesenchymal transdifferentiation and organ fibrosis 40,41. The present study found that plakoglobin was associated with p53 and TCF-4 in HCAECs (Figure 10C). KLK8 overexpression led to a significant increase in the association of plakoglobin with both p53 and TCF-4 (Figure 10C). In addition, KLK8-induced EndMT was significantly reduced in the presence of the p53 inhibitor pifithrin-α (Figure S10A-B). However, ICG-001, an inhibitor of β-catenin/TCF-mediated transcription, had no significant effect on KLK8-induced EndMT (Figure S10D-E). These results suggest that plakoglobin is required for KLK8-induced EndMT by cooperating with the p53 signaling pathway.

As aforementioned, the present results indicated that increased TGF-β1 production may be the cause of KLK8-induced EndMT (Figure 7G-I). The present study next investigated the mechanistic link between KLK8/plakoglobin/p53 signaling pathway and TGF-β1 production. It was found that the KLK8-induced increase in TGF-β1 mRNA expression was significantly attenuated in the presence of plakoglobin siRNA or the p53 inhibitor pifithrin-α (Figure 10D and Figure S10C). However, ICG-001 treatment had no significant effect on KLK8-induced TGF-β1 expression (Figure S10F). These results suggest that KLK8-mediated plakoglobin nuclear translocation may lead to upregulation of TGF-β1 expression at the transcriptional level via a p53-dependent pathway. Next, transcriptional factor motif searches in TGF-β1 promoter were performed using the P-match program. Unexpectedly, the program predicted no p53 consensus binding site in the region located 20-kb upstream of the human TGF-β1 coding sequences. Previous studies indicate that hypoxia inducible factor (HIF)-1α can directly bind to the hypoxia response element of the TGF-β1 promoter, thus driving TGF-β1 gene expression 42. Notably, p53 and HIF-1α have been found to functionally cooperate in hypoxia-induced gene transcription 43. Using ChIP analysis, the present study confirmed the binding of HIF-1α to the TGF-β1 promoter, which was significantly enhanced by KLK8 overexpression (Figure 10E). The HIF-1α inhibitor echinomycin not only inhibited basal TGF-β1 expression, but also blocked KLK8-induced TGF-β1 mRNA expression and release in HCAECs (Figure 10D). KLK8 overexpression induced the association of p53 with HIF-1α, which was blocked by plakoglobin knockdown (Figure 10F). In addition, the KLK8-induced HIF-1α binding to the TGF-β1 promoter was largely blocked in the presence of plakoglobin siRNA or the p53 inhibitor pifithrin-α (Figure 10E). Altogether, these data suggest that upregulation of KLK8 leads to plakoglobin-dependent association of p53 with HIF-1α, which further enhances the transactive effect of HIF-1α on the TGF-β1 promoter.

p53 has been implicated in the transcriptional regulation of TGF-β1 target genes via cooperation with Smad proteins 44,45. The present study found that KLK8 overexpression significantly increased the association of p53 with Smad3, which was blocked by plakoglobin knockdown (Figure 10F). Ad-KLK8-treated HCAECs exhibited profoundly elevated mRNA levels of pro-EndMT target genes of the TGF-β1/Smad signaling pathway, including Snail, Slug, Zeb1, Zeb2 and Twist 46, compared with those of Ad-vector-treated HCAECs (Figure 10G-H). Moreover, the KLK8-induced increase in mRNA expressions of pro-EndMT target genes was significantly reduced in the presence of plakoglobin siRNA (Figure 10G) or the p53 inhibitor pifithrin-α (Figure 10H). Taken together, these data highlight the role of KLK8-induced cooperation of p53 with Smad3 in promoting pro-EndMT reprogramming by TGF-β1 in endothelial cells.

### High glucose promotes plakoglobin-dependent cooperation of p53 with HIF-1α and Smad, subsequently increasing the expression of TGF-β1 and its pro-fibrotic target genes in a KLK8-dependent manner

**Figure 11:**
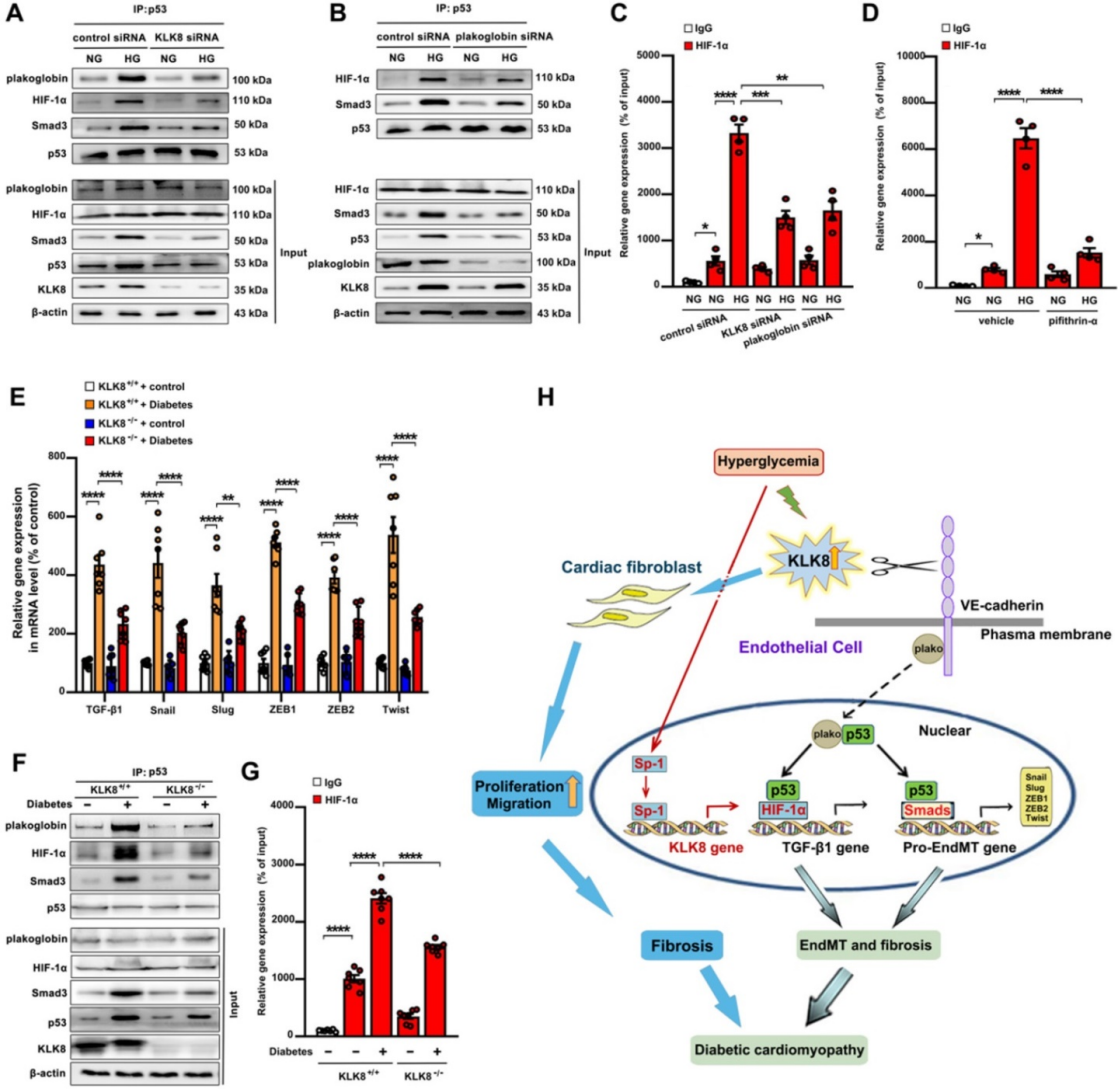
High glucose promotes plakoglobin-dependent cooperation of p53 with HIF-1α and Smad, subsequently increasing the expression of TGF-β1 and its pro-EndMT target genes in a KLK (kallikrein-related peptidase) 8-dependent manner. A, Immunoprecipitation assay showed that high glucose (HG, 25 mM glucose) treatment enhanced the association of p53 with plakoglobin, HIF-1α and Smad3 in HCAECs, which was reduced by KLK8 knockdown. B, Immunoprecipitation assay showed that HG-induced association of p53 with HIF-1α and Smad3 was reduced by plakoglobin knockdown in HCAECs. C and D, ChIP assay showed that the HG-induced HIF-1α binding to TGF-β1 promoter was blocked by knockdown of KLK8 or plakoglobin (C) or p53 inhibitor pifithrin-α (D, 20 μM). Data are expressed as means ± SEM (n = 4). NG indicates normal glucose. E-G, Heart tissues were obtained from the KLK8-deficient (KLK8^-/-^) and KLK8^+/+^ mice after 24 weeks of diabetes. The mRNA levels of the pro-EndMT target genes of TGF-β1/Smad pathway (Snail, Slug, Zeb1, Zeb2 and Twist) were decreased in the hearts obtained from KLK8^-/-^ diabetic mice, as compared to KLK8^+/+^ diabetic mice (E). F, Immunoprecipitation assay showed that the association of p53 with plakoglobin, HIF-1α and Smad3 was reduced in KLK8^-/-^ diabetic mice as compared to KLK8^+/+^ diabetic mice. G, ChIP assay showed that KLK8 deficiency suppressed HIF-1α binding to TGF-β1 promoter in diabetic myocardium. Data are expressed as means ± SEM (n = 7). *p < 0.05, **p < 0.01, ***p < 0.001, ****p < 0.0001. H, Schematic diagram of the mechanism by which KLK8 promotes EndMT and cardiac fibrosis in diabetic cardiomyopathy. Hyperglycemia upregulates KLK8 expression in endothelial cells, which cleavages the VE-cadherin extracellular domain and promotes plakoglobin nuclear translocation and its cooperation with p53. The plakoglobin-dependent cooperation of p53 with HIF-1α and Smad3 subsequently increased the expression of TGF-β1 and the pro-EndMT target genes of TGF-β1/Smad pathway, which finally promotes the differentiation of endothelial cells into mesenchymal cells and the pathogenesis of cardiac fibrosis.

As shown in supplemental Figure S11A-B, high glucose treatment resulted in a significant loss of membrane and cytosolic plakoglobin, whereas it caused a marked nuclear translocation of plakoglobin. KLK8 knockdown reduced nuclear plakoglobin levels, whereas it increased membrane and cytosolic plakoglobin levels in high glucose-treated HCAECs. It was found that high glucose treatment resulted in a significant increase in TGF-β1 mRNA expression and release, as well as a significant increase in the mRNA levels of TGF-β1 pro-EndMT transcriptional targets, which were largely reduced by siRNA targeting KLK8 and plakoglobin (Figure 4E, Figure S11C-D). Activation of the p53 and Smad signaling pathways has been implicated in the pathogenesis of high glucose-induced endothelial dysfunction and EndMT, respectively 13,14,47. As shown in Figure 11A, it was found that high glucose led to a significant increase in the association of plakoglobin with p53, which was blocked by KLK8 knockdown. In addition, high glucose induced p53 binding to both HIF-1α and Smad3, which was blocked by siRNA targeting KLK8 and plakoglobin (Figure 11A-B). Using ChIP analysis, it was found that high glucose treatment led to a significant increase in the binding of HIF-1α to the TGF-β1 promoter, which was largely blocked in the presence of KLK8 siRNA, plakoglobin siRNA (Figure 11C) or the p53 inhibitor pifithrin-α (Figure 11D).

Using an STZ-induced diabetes model, a significant increase in the TGF-β1 mRNA and protein levels, as well as in the mRNA levels of TGF-β1 pro-EndMT transcriptional targets, was observed in heart tissues after 24 weeks of diabetes mellitus, which was significantly attenuated in KLK8^-/-^ mice (Figure 2F, Figure 11E). The association of plakoglobin with p53 in diabetic heart tissues was decreased in KLK8^-/-^ mice (Figure 11F). Diabetes mellitus increased the association of p53 with both HIF-1α and Smad3 in heart tissues, which was attenuated in KLK8^-/-^ mice (Figure 11F). Using ChIP analysis, it was found that KLK8^-/-^ mice exhibited less binding of HIF-1α to the TGF-β1 promoter in diabetic heart tissues compared with that of KLK8^+/+^ mice (Figure 11G). Taken together, these *in vitro* and *in vivo* results suggest that high glucose may promote plakoglobin-dependent cooperation of p53 with HIF-1α and Smad3, subsequently increasing the expression of TGF-β1 and its pro-EndMT target genes in a KLK8-dependent manner (Figure 11H).

## Discussion

It is known that both cardiac hypertrophy and fibrosis are common structural hallmarks of patients with diabetes, resulting in cardiac stiffness and impaired cardiac function 6. The present study reports for the first time that diabetes mellitus-associated EndMT and fibrosis in the myocardium are stimulated in part by upregulation of KLK8. Degradation of VE-cadherin and activation of the plakoglobin-dependent pro-EndMT signaling pathway by KLK8 contribute to the development of EndMT and cardiac fibrosis and accelerate the progression to cardiac dysfunction in diabetes mellitus.

A previous study reported that high glucose stimulates the expression of KLK1, 5 and 6 in HAECs, and the combination of these three KLKs and other two serine proteases (elastases 1 and 2) induces EndMT *in vitro* 20. However, whether other KLK family members are involved in high glucose-induced EndMT during the development of diabetic cardiac fibrosis remains unknown. The present study found that KLK8 was the highest induced KLK in diabetic myocardium. Global deletion of KLK8 significantly alleviated diabetes mellitus-induced EndMT and cardiac fibrosis, whereas transgenic overexpression of KLK8 led to interstitial/perivascular fibrosis and EndMT in the myocardium. *In vitro* studies revealed that KLK8 knockdown significantly alleviated high glucose-induced EndMT, whereas KLK8 overexpression in endothelial cells was sufficient to induce the process of EndMT. Collectively, these findings suggest that upregulation of KLK8 contribute to the development of diabetes mellitus-induced EndMT and cardiac fibrosis. Of note, since KLK8 promotes proliferation and migration in cardiac fibroblasts, a potential contribution of KLK8-induced fibroblast activation to the development of cardiac fibrosis in the context of diabetes cannot be excluded (Figure 11H).

Abnormal lipid metabolism has been implicated in the pathogenesis of diabetes-associated organ fibrosis 48,49*.* For example, increased FFA release from adipose tissue and high cholesterol contribute to the development of diabetic cardiac fibrosis and liver fibrosis, respectively 50,51. Recently, Li *et al* reported that the kallikrein gene cluster (KLK1/3/7/8/12) is upregulated >17-fold in patients with high altitude-associated polycythemia (HAPC) 52. As hypertension is an important clinical symptom of HAPC and is closely associated with abnormal cholesterol metabolism, the interaction between KLK members and cholesterol was assessed by AutoDock analysis. Cholesterol tended to bind sites located near the C-terminus of KLK1 and KLK2, the N-terminus of KLK7 and KLK12, and the middle region of the KLK8 sequence, suggesting that KLK members may directly interact with cholesterol and contribute to the regulation of cholesterol metabolism. Regarding the regulatory effect of KLK members on FFA metabolism, a recent study demonstrated that the ablation of KLK7 in adipose tissue led to a slight, but not significant decline in serum FFA levels in both normal chow and HFD-treated mice 53. The present study found that KLK8 deficiency significantly attenuated diabetes-induced increases in the plasma levels of TC and FFA. However, the precise mechanism by which KLK8 regulates lipid metabolism awaits future experiments.

A major implication of high glucose-induced KLK8 upregulation is that KLK8-targeted therapy would have clinical relevance for patients with diabetic myopathy. In fact, increased levels of KLK8 have been implicated in the pathogenesis of various diseases including psoriasis 54, schizophrenia, mood and anxiety disorders 15,55, Alzheimer's disease 56,57 and several types of cancers 58-60. The critical role of KLK8 in this wide spectrum of diseases motivated the development and evaluation of KLK8-targeted therapies. To date, a number of different pharmaceutical approaches, including peptide-based and small molecule inhibitors, macroglobulins, engineered natural inhibitors, noncovalent inhibitors, and antibodies, have been used in the development of KLK8-targeted therapy 60-62. However, the majority of them were demonstrated to be efficient in blocking KLK8 activity *in vitro*. Notably, recent preclinical studies by Herring and the colleagues demonstrated that antibody-mediated KLK8 inhibition improves neuroplasticity and memory, reduces fear and attenuates Alzheimer's disease pathology in mice 56,57. These findings prompt us to consider the use of antibody-mediated blockade of KLK8 as a promising therapeutic approach against diabetes myopathy, which merits further investigation.

KLK8 is originally cloned as neuropsin in the mouse brain. During development, KLK8 mRNA is expressed abundantly in the developing heart, lung, pituitary, choroid plexus, epithelial lining of the skin and the central nervous system. Despite such a broad distribution, KLK8 knockout mice are born and develop without gross defects 63. In consistence with these literatures, the present study demonstrated that KLK8 deletion had no significant effects on cardiac function, glucose metabolism and lipid metabolism in healthy animals. Given that the epidermis of the skin and brain are two of the tissues that exhibit high level of KLK8 expression in physiological conditions, current studies mainly focus on the physiological or pathological roles of KLK8 in these two organs. It is found that KLK8 knockout mice exhibit reduced skin hyperkeratosis and acanthosis following skin stimulation with sodium lauryl sulfate. However, morphological analysis shows that there is no apparent difference in the epidermis of unstimulated KLK8 knockout mice compared with unstimulated wild-type mice 64. There is no apparent difference between wild-type and KLK8 knockout mice for depressive-like behaviour under baseline conditions 65. On the other hand, KLK8 deficient mice display loss of neurogenesis and synaptogenesis in the hippocampus, and are unable to regulate physiological synaptic plasticity and suffer from long-term potentiation (LTP) deficits and frail memory acquisition 66,67. Notably, recent studies demonstrate that the memory performance of KLK8 antibody-treated healthy mice deteriorates significantly as compared with IgG-treated healthy mice. In addition, cognitive impairments are accompanied by a tendency to hyperactivity 56,57. Taken together, these findings indicate that although KLK8 inhibition may become an effective strategy against diabetic myopathy, hyperkeratotic skin disease and Alzheimer's disease, it must be used with great caution due to its central nervous system side effects.

Due to the high expression levels of KLK8 (also named neuropsin) in the brain, the proteolytic function of KLK8 has been investigated in the central nervous system (CNS) 68-70. As a secreted serine protease, KLK8 is known to cleave the extracellular portion of several membrane proteins. The KLK8-mediated cleavage of neuregulin-1, synaptic adhesion molecule L1, and ephrin type-B receptor 2 has been implicated in the regulation of hippocampal neural plasticity and the pathogenesis of anxiety disorders 68-70. However, whether the proteolytic function of KLK8 contributes to extra-CNS diseases remains largely unknown. A previous study has shown that KLK8 overexpression can promote cardiomyocyte hypertrophy through epidermal growth factor and protease-activated receptor-dependent pathways 19. Here, we provided multiple lines of evidence that KLK8 was critically involved in the proteolytic processing of VE-cadherin, a major adhesive protein of inter-endothelial junctions 35.

Ectodomain shedding of VE-cadherin in the vasculature is of critical importance for vascular biology. Members of the matrix metalloproteinases (MMPs), a disintegrin and metalloproteinases (ADAMs) and elastase families, have been found to mediate the proteolytic degradation of VE-cadherin, thus playing critical roles in the regulation of angiogenesis, endothelial cell migration and endothelial barrier function 71-73. In particular, MMP-mediated proteolytic degradation of VE-cadherin has been reported to be an important mechanism for the loss of the blood-retinal barrier in diabetic retinopathy 74. The present study additionally contributed to the understanding of this complex system, demonstrating that KLK8 mediated the ectodomain shedding of VE-cadherin, which may represent a potent mechanism for the initiation of EndMT in the myocardium during the pathogenesis of diabetes mellitus-induced cardiac fibrosis.

VE-cadherin forms a complex with p120-catenin, β-catenin and plakoglobin to stabilize adheren junctions in endothelial cells 35. In endothelial adherens junctions, β-catenin and plakoglobin bind directly to the cytoplasmic tail domains of VE-cadherins 35,75. While cadherin-bound β-catenin and plakoglobin are required for cell adhesion, membrane-uncomplexed β-catenin and plakoglobin have a function in transducing the Wnt signal from the cell surface to the nucleus 39,76,77. These proteins finally interact with TCF and lymphoid enhancer factor (LEF) to regulate the expression of canonical Wnt target genes 39,76,77. In addition to interacting with TCF/LEF transcription factors, plakoglobin also regulates the expression of various genes involved in tumorigenesis and metastasis by interacting with p53 and associating with the promoter of the 14-3-3σ gene 38. Previous study has shown that MMP-7 degrades VE-cadherin and releases β-catenin from the VE-cadherin/catenin complex, thereby accelerating the nuclear accumulation of β-catenin in human umbilical vein endothelial cells 36. The present study contributed to the understanding of this complex system, demonstrating that KLK8 was capable of degrading VE-cadherin, thus promoting the nuclear translocation of plakoglobin. In addition, plakoglobin was indispensable for the induction of EndMT by KLK8. Notably, although KLK8 overexpression led to a significant increase in the association of plakoglobin with both p53 and TCF-4, KLK8-induced EndMT was reduced only by a p53 inhibitor, but not by the inhibitor of β-catenin/TCF-mediated transcription. These findings suggest that plakoglobin is required for KLK8-induced EndMT by cooperating with the p53 signaling pathway.

Elevated myocardial levels of p53 have been reported in diabetic patients, diabetic animal models and in high glucose-treated cardiomyocytes 78-80. Global p53 deficiency protects against cardiac metabolic abnormalities in diabetic mellitus 79. Inhibition of p53 prevents diabetes mellitus-induced cardiac fibrosis and dysfunction by preventing cell senescence, reduced glycolysis, and impaired angiogenesis 81. Although the specific role of endothelial p53 in the development of diabetic cardiomyopathy remains unknown, endothelial p53 deletion has been demonstrated to inhibit mesenchymal differentiation, and to prevent cardiac fibrosis and heart failure induced by pressure overload 82. In an *in vitro* study, lentivirus-mediated overexpression of p53 induced EndMT by activating TGF-β/Smad3, PI3K/Akt/mTOR and MAPK/ERK signaling in a TGF-β-dependent manner 83. These findings suggest a causal role of p53 in the development of EndMT and cardiac fibrosis in diabetic mellitus. Previous study has found that plakoglobin interacts with p53 and increases its transcriptional activity, thereby regulating the expression of various genes involved in tumorigenesis and metastasis 38. The present study found that upregulation of KLK8 leads to plakoglobin-dependent nuclear translocation of p53, which binds to HIF-1α, and further enhances the transactivation effect of HIF-1α on the TGF-β1 promoter and consequently upregulates TGF-β1 expression at the transcriptional level. In addition, KLK8-induced p53 activation promoted the pro-fibrotic reprogramming by TGF-β1 via the cooperation of p53 with Smads in endothelial cells. The present study identified KLK8-mediated plakoglobin nuclear translocation as a new modulator of p53-mediated induction of the TGF-β1 signaling pathway, which is able to promote the EndMT process in the context of diabetic cardiomyopathy.

In conclusion, the present study provides several lines of evidence supporting a new pro-EndMT mechanism during the pathogenesis of diabetic cardiac fibrosis via the upregulation of KLK8. KLK8-induced EndMT is mediated by cleavage of the VE-cadherin extracellular domain, which promotes plakoglobin nuclear translocation and its cooperation with p53. High glucose may promote the plakoglobin-dependent cooperation of p53 with HIF-1α and Smad3, subsequently increasing the expression of TGF-β1 and the pro-EndMT target genes of the TGF-β1/Smad signaling pathway in a KLK8-dependent manner both *in vitro* and *in vivo*. Such findings may serve for the development of potential future KLK8-based therapeutic strategies for the treatment of diabetic cardiomyopathy.

## Methods

### Animal models

All laboratory mice and rats in this study were maintained in a pathogen-free facility at the Animal Research Center of Navy Medical University. Animal studies (mice and rats) were performed in accordance with the Guide for the Care and Use of Laboratory Animals published by the NIH (NIH publication No. 85-23, revised 1996), and were approved by the Ethics Committee of Navy Medical University. Animal experiments were randomized with a random numbers table. All *in vivo* studies were blinded for both genotype and treatment during the measurement and analysis stages. Of note, only male mice were included in all *in vivo* experiments in this study.

The present study used KLK8 transgenic rats generated by our research group 19. PCR based assays, using the genomic DNA extracted from tail tip biopsies as a template, were used to identify transgenic strains according to the previously described protocol 19.

The KLK8-flox mouse line was generated at the Shanghai Biomodel Organism Science & Technology Development Co., Ltd (Shanghai, China) using a LoxP targeting system with two LoxP elements flanking exon 1-3 of KLK8. Briefly, the two LoxP elements were inserted into the KLK8 gene by homologous recombination in embryonic stem (ES) cells. The positive homologous recombinant ES cell clone was then injected into blastocyst of C57BL/6J mice to obtain chimeric mice, which were mated with Flp mice to delete Neo cassette and create heterozygous F1 mice with confirmed loxP-sites. To generate global KLK8 knockout mice, KLK8-flox mice were mated with EIIa-Cre transgenic mice (The Jackson Laboratory). Deletion of the KLK8 gene was verified by PCR of genomic DNA using (5'-GGACGTTGGAGTCACAGC-3') and (5'-CCCAGGAGCAGAAGAGTG-3') primers. KLK8^flox/flox^; EIIa-Cre(+) mice (KLK8^-/-^), along with age-matched KLK8^flox/flox^; EIIa-Cre(-) littermates as controls, were used to investigate the impacts of KLK8 deficiency.

Mice with the same genotype were assigned to 1:1 allocation ratio to control and diabetic groups. Diabetes was induced by the intraperitoneal injection of 100 mg/kg streptozotocin (STZ, Sigma Aldrich, St. Louis, MO) in citrate buffer (pH 4.5) for 2 consecutive days as described previously 13,84,85. Mice with nonfasting glucose levels of ≥ 250 mg/dL on day 4 after injection were considered diabetic.

### Cell culture and high glucose treatment

Cryopreserved human coronary artery endothelial cells (HCAECs) were purchased from Lonza (Walkersville, MD). HCAECs were cultured in Endothelial Cell Growth Basal Medium-2 (EBM-2, Lonza) containing normal glucose (NG, 5.5 mM D-glucose) in a humidified 5% CO_2_ incubator at 37 ℃ and used between the fourth and sixth passages. To observe the effect of high glucose on the expression of KLK8 and EndMT markers, HCAECs were either maintained in NG EBM-2 (plus 19.5 mM mannitol to control for osmolality) or switched to high glucose (15 mM D-glucose plus 10 mM mannitol, or 25 mM D-glucose ) EBM-2. The medium was changed every 48 h for 5 consecutive days.

### Echocardiography

Mice were anaesthetized with isoflurane (2.5%) via inhalation. For each measurement, a B-mode image was first obtained to make sure the appropriate positioning of the scan head. Then, transthoracic M-mode echocardiographic recordings were performed using an Esaote MyLab Touch system (Esaote Biomedica, Genova, Italy) ultrasound machine 19,86. We measured the diastolic diameter (Dd) and systolic diameter (Ds) of the left ventricle under the long-axis parasternal views. Interventricular septum thickness (IVS), left-ventricular end-diastolic and -systolic internal diameter (LVEDD and LVESD, respectively), left-ventricular end-diastolic posterior wall thickness (LVPWd) and left-ventricular end-diastolic and -systolic volum (LVEDV and LVESV, respectively) were measured. Fractional shortening (FS) and ejection fraction (EF) were calculated using the Teichholz method 86. These parameters were calculated as follows: LVEDV (ml) = 7×(Dd^3^/(2.4+Dd); LVESV (ml) = 7×Ds^3^/(2.4+Ds; EF (%) = (LVEDV-LVESV) / LVEDV ×100% and FS (%) = (Dd-Ds)/Dd×100%. All measurements represented the mean values of the signals from three consecutive cardiac cycles.

### Mice blood pressure detection

A noninvasive, computerized tail-cuff system (ALC-NIBP, ALCBIO; Shanghai, China) was used to measure the blood pressure in mice. Briefly, mice were first acclimated to restraint, tail-cuff blood pressure measurements for 3 consecutive days, and blood pressure was then measured on the fourth day. The values of blood pressure were recorded when five consecutive stable readings were available. The highest and lowest readings were discarded, and the remaining three readings were averaged for one data point 87,88.

### Immunohistochemistry

Immunohistochemistry (IHC) was performed as previously described 19. Paraffin heart sections (5 μm) were stained with anti-KLK8 antibody (Santa Cruz, sc-67666). For negative controls, the primary antibody was substituted with a goat IgG in the same dilution. Staining was independently assessed by two experienced pathologists blinded to group allocation 89. The score for KLK8 staining was based on the integrated staining intensity and the proportion of positive cells. Staining intensity was scored as follows: 0 = no color; 1 = yellow; 2 = light brown; and 3 = dark brown. The proportion of KLK8-positive cells (number of positively labeled cells / number of total cells) was scored as follows: 0, positive cells <10%; 1, 10%-40% positive cells; 2, 40%- 70% positive cells; and 3, positive cells ≥70%. The final score was determined by adding the staining intensity score and average proportion of positive cells score. All the KLK8 staining scores were expressed as the average of six randomly selected microscopic fields.

### Masson's trichrome staining

Paraformaldehyde (4%) fixed hearts were sectioned transversely at the mid-ventricular level, embedded in paraffin. Five-μm-thick sections were generated, stained with Masson's trichrome staining (Servicebio, Wuhan, China) according to the manufacturer's instructions 90, and then examined by light microscopy. For each slide, the percentage of fibrotic area was quantified by manually tracing the blue area in five randomly selected microscopic fields using Image-Pro Plus software 6.0 (Media Cybernetics, Bethesda, MD, USA) 90. The investigator performing fibrotic area analysis was blinded to group allocation.

### Primary neonatal rat cardiac fibroblasts isolation

Primary neonatal rat cardiac fibroblasts were isolated using a modified method as previously described 91,92. Briefly, ventricle tissues of Sprague Dawley neonatal rats (1-3 days old) were collected and minced into 1 mm^3^ pieces then were digested in 0.25% trypsin for 6 min at 37 °C with gentle shaking. The digestion was repeated five times to obtain single cells, and the supernatant from the first digestion was discarded. Cells from the subsequent digestions were placed in DMEM supplemented with 10% fetal bovine serum and centrifuged. The cells were resuspended in DMEM with 10% fetal bovine serum, and were plated into a culture incubator (37 °C in 5% CO_2_-95% air) for 1.5 h for purification. Then, the suspension was discarded, and the sediment (cardiac fibroblasts) was maintained and cultured in the incubator.

### Subcellular protein fractionation and Western Blot analysis

For the total protein extract preparation, heart tissues or HCAECs or primary neonatal rat cardiac fibroblasts were lysed with chilled RIPA lysis buffer (Beyotime) with 1 × Protease Inhibitor Cocktail (Sigma-Aldrich). Nuclear, cytoplasmic, and plasma membrane fractions of cellular extracts were prepared by using a subcellular protein fractionation kit for cultured cells (Thermo Fisher) according to the manufacturer's instructions. Protein samples were then subjected to 10% SDS-PAGE, and transferred to polyvinylidene fluoride (PVDF) membranes. The membrane was blocked with 5% skim milk powder in Tris-buffered saline containing 0.1% Tween 20, then incubated with primary antibodies against VE-cadherin (Santa Cruz, sc-52751), CD31 (Abcam, ab222783; Proteintech, 11265-1-AP), plakoglobin (Santa Cruz, sc-7900), vimentin (Santa Cruz, sc-6260), α-SMA (Santa Cruz, sc-32251), KLK8 (Santa Cruz, sc-67666), integrin β3 (Santa Cruz, sc-365679), p53 (Proteintech, 10442-1-AP), TCF-4 (Santa Cruz, sc-166699), Smad3 (Santa Cruz, sc-101154), histone H3 (Santa Cruz, sc-517576), kinin B_1_ receptor (Santa Cruz, sc-25484), kinin B_2_ receptor (Santa Cruz, sc-25671), Ki67 (Abcam, ab16667), PCNA (Santa Cruz, sc-25280), Sp-1 (Santa Cruz, sc-59), GAPDH (Cell Signaling Technology, Beverly, MA, USA, #2118), or β-actin (Santa Cruz, sc-47778), then incubated with secondary antibodies for 1 h at room temperature. Chemiluminescence was performed with Western blotting detection system (Santa Cruz). The chemiluminiscent signal from the membranes was quantified by a GeneGnome HR scanner using GeneTools software (SynGene, San Diego, CA).

### Quantitative Real-time RT-PCR

Total RNA from myocardium or HCAECs was extracted by a TRIzol reagent (Takara), and then 2 ug RNA was reverse transcribed to generate cDNA by using superscript reverse Transcriptase (Invitrogen). Quantitative real-time PCR was carried out using a MiniOpticon real-time PCR detection system (Bio-Rad Laboratories). The reaction solution consisted of 2.0 μL diluted cDNA, 0.2 μM of each paired primer, 200 μM deoxynucleotide triphosphates, 1 U Taq DNA polymerase (Qiagen, Beijing, China), and 1×PCR buffer. SYBRGreen (Roche Ltd., Basel, Switzerland) was used as detection dye. The annealing temperature was set at 58-62 °C and amplification was set at 40 cycles. The temperature range to detect the melting temperature of the PCR product was set from 60 to 95 °C. To determine the relative quantitation of gene expression, the comparative Ct (threshold cycle) method with arithmetic formulae (2-^△△^Ct) was used. Messenger RNA levels were normalized relative to the house-keeping gene β-actin. The primer sequences used in this study were provided in Table S2.

### 3-[4,5-Dimethylthiazol-2-yl]-2,5-diphenyl tetrazolium bromide (MTT) assay

Cell viability was evaluated by MTT assay based on the reduction of MTT (Sigma-Aldrich) by functional mitochondria to formazan, as described previously 93.

### Measurement of hydroxyproline content

Heart tissues were homogenized in cold PBS containing proteinase inhibitor cocktail (Sigma-Aldrich). The hydroxyproline content in heart tissue was determined according to the kit manufacturer's instructions (Winching, Nanjing, China).

### Measurement of insulin, thrombomodulin, von Willebrand factor, E-selectin, total cholesterol, triglyceride, free fatty acid, high-density lipoprotein cholesterol, low-density lipoprotein cholesterol, collagen I and TGF-β1 contents

Heart tissues were homogenized in cold PBS containing proteinase inhibitor cocktail (Sigma-Aldrich). The contents of collagen I and TGF-β1 in heart tissues, the contents of insulin, thrombomodulin, von Willebrand factor and E-selectin in plasma, the contents of thrombomodulin, von Willebrand factor, E-selectin and TGF-β1 in the supernatants of HCAECs cell cultures were assessed by commercially available ELISA kits obtained from Lifespan Bioscience (Seattle, WA, USA). Plasma concentrations of total cholesterol (TC), triglyceride (TG), free fatty acid (FFA), high-density lipoprotein cholesterol (HDL-C) and low-density lipoprotein cholesterol (LDL-C) were measured using an automatic biochemistry analyzer (Hitachi 7150, Tokyo, Japan). The contents of collagen I and TGF-β1 in heart tissues were calculated as concentrations per μg protein.

### Chromatin Immunoprecipitation asssay

Chromatin Immunoprecipitation (ChIP) analysis from heart tissues and HCAECs samples was performed with EZ-ChIP^TM^ kit (Millipore, Billerica, MA) by using HIF-1α antibody (Proteintech, 20960-1-AP; Santa Cruz, sc-13515) and immunoglobulin (Ig) G, according to the manufacturer's instructions. Briefly, crosslinked chromatin was sonicated to about 200 to 1000-bp fragments by enzymatic digestion. The chromatin was immunoprecipitated with primary antibody against HIF-1α overnight and then incubated with 60 uL protein G beads for 1 h at 4 °C with rotation. IgG was served as the negative control. After washing, elution and de-crosslinking, the co-precipitated DNA was analyzed by quantitative PCR using specific primers, 5′-AAGGCACAGAGGGAGTCGTTGTCC-3′ and 5′-AAGAGGAGGTGGCGAGTGAGAGGC-3′, to the HIF1-α-binding region of the TGF-β1 promoter 42.

### Infection of adenovirus and lentivirus

KLK8 adenovirus was generated by using the AdEasyTM adenoviral vector system (Stratagene, La Jolla, CA, USA) as previously described 19. Lentivirus expressing VE-cadherin was constructed by Genechem (Shanghai,China). The cell infection was performed according to the manufacturer's protocol.

### Transfection of Small interfering RNA (siRNA)

The siRNAs for KLK8 and plakoglobin were designed and synthesized by GenePharma Corporation (Shanghai, China). The target sequences for human KLK8 siRNA and plakoglobin siRNA are as follows: 5'-TGGAGGACCACAACCATGATCTGAT-3' and 5'-CCCTCGTGCAGATCATGCGTAACTA-3', respectively. Negative control siRNA was scrambled sequence without any specific target: 5'-TTCTCCGAACGTGTCACGT-3'. Transfection of siRNA in HCAECs was performed by using the Xfect^TM^ RNA transfection reagent (Takara) according to the manufacturer's instructions.

### FITC-dextran assay

FITC-dextran (molecular weight 10 kDa) was used to detect endothelial permeability *in vitro* as previously described 94 HCAECs were grown to confluence in Transwell inserts (0.4 μM, 12-mmdiameter, Corning, Corning, NY, USA). After treatment of the cells, medium containing 10-kDa FITC-dextran with a final concentration of 1 mg/mL was added in the top chamber of the Transwell. 1 h later, the amount of FITC-dextran that permeated the endothelial monolayer into the lower compartment was measured using a Synergy fluorescence plate reader (Bio-Tek, Cytation3, USA; excitation 485±20 nm, emission 528±20 nm).

### Immunoprecipitation

HCAECs or heart tissues were lysed with cold RIPA lysis buffer (Beyotime, China) containing 1% Proteinase Inhibitor Cocktail (Sigma-Aldrich) and proteasome inhibitor (N-ethylmaleimide, at 20 mM). Cell lysates were incubated on ice for 2 h, and cell debris was removed by centrifugation. The clarified supernatants were incubated with antibodies against KLK8 (Abcam ab150395, Santa Cruz sc-292341), p53 (Proteintech, 10442-1-AP), or plakoglobin (Santa Cruz, SC-7900) at 4 °C for 16 h with gentle rotation. IgG was used as control for nonspecific interaction. Protein A and G sepharose beads (Beyotime) were added and incubated at 4 °C for an additional 3 h, and the immune complexes were washed four times. The final precipitate was boiled in a protein loading buffer for 5 min and eluted on 10% SDS-PAGE for Western blot analysis using the respective antibodies.

### Mass Spectrometry and Bioinformatics Analysis

Protein was extracted from the myocardium of KLK8 transgenic rats and immunoprecipitated with the primary antibody against KLK8. The immunoprecipitates were separated by SDS-PAGE and stained with the Colloidal Blue Staining kit (Beyotime). Each gel lane was cut into pieces followed with trypsin digestion, and peptide flagments were then extracted and identified via ultra performance liquid chromatography trandem mass spectrometry (UPLC-MS/MS) (Bioclouds, Shanghai, China) 95-97. Briefly, digestion of gel pieces was performed with trypsin buffer at 37 °C for 12 h, followed by the addition of formic acid [0.1% (v/v) final] to acidify the tryptic peptides. Then, peptide samples were separated with the nano ACQUITY UPLC (Waters Corporation, Milford) and detected with the Q Exactive hybrid quadrupole-Orbitrap mass spectrometer (Thermo Fisher Scientific). The MS/MS spectra were preprocessed with PEAKS studio version 8.5 (Bioinfor Inc., CA) and the PEAKS DB was searched against the Rattus database (UniProtKB/Swiss-Prot), assuming the digestion enzyme Trypsin. The following search parameters were used: Fixed modifications: Carbamidomethyl (C); Acetylation (Protein N-term), Deamidation (NQ), Variable modifications: Oxidation (M); Missed cleavages: 2; MS mass tolerance: ± 10.0 ppm; MSMS mass tolerance: ± 0.02 Da.

### N-terminal protein sequencing

HCAECs were exposed to KLK8 adenovirus for 72 h in serum-free culture media. The supernatant was collected and then concentrated by ultrafiltration (10 kD molecular weight cut-off, Millipore, Millipore, Bedford, MA). Samples were then separated by SDS-PAGE, and transferred to a PVDF membrane. The membrane was stained with Coomassie blue staining solution. The ~30 kDa band was cut out from the membrane and collected. N-terminal sequencing was then performed using the Edman degradation method at Biotech Pack Scientific (Beijing, China) on a PPSQ-33A system from Shimadzu (Kyoto, Japan).

### Determination of KLK8 promoter activity

The genomic DNA fragment upstream from the transcriptional start site of the human KLK8 gene, containing the Sp-1 site, was synthesized and cloned into pGL3-basic plasmid (pGL3-KLK8) by Generay Biotech Co. To construct the Sp-1 site mutated plasmid pGL3-KLK8ΔSp-1, the Sp-1 response element (5'-GGCGG-3') in the pGL3-KLK8 plasmid was mutated to 5'-TTCGT-3' (Generay Biotech Co) 98,99. HCAECs were seeded into a 48-well culture plate and incubated at 37 °C in 5% CO_2_-95% air. After 18 h of incubation, the cells were transfected with the above pGL3-luciferase reporter plasmids (100 ng/well) and pRL-TK-Renilla luciferase plasmid (10 ng/well; Promega) using Lipofectamine 3000 (Invitrogen). The cells were then treated with high glucose (25 mM). Luciferase assays were performed 48 h later using the dual-luciferase assay kit (Promega). Relative luciferase activity is presented as firefly luciferase values normalized to renilla luciferase activity 100.

### Cell proliferation assays

HCAECs were seeded in 48-well plates (200 μL/well) at the density of 1 × 10^5^ cells/ml for 24 h. Cell viability was determined at 72 h after KLK8 adenovirus infection using a Cell Counting Kit-8 (CCK8, Beyotime, China) according to manufacturer's instructions. The absorbance of each well were measured and obtained the OD values at 490 nm with a microtiter plate reader (BioTek, USA).

### Immunofluorescence analysis

Paraffin sections (5 μm) of heart tissues were rehydrated, and microwaved in citric acid buffer to retrieve antigens. After incubation with 10% BSA for 1 h, the sections were incubated with primary antibodies against CD31 (Abcam, ab222783), KLK8 (Santa Cruz, sc-67666), α-SMA (Santa Cruz, sc-32251), vimentin (Santa Cruz, sc-6260), and FSP-1 (Santa Cruz, sc-100784) at a dilution of 1:100 at 4 ºC overnight. After washes, sections were incubated with Alexa Fluor 568-conjugated anti-rabbit IgG (Invitrogen, Carlsbad, CA) for CD31 and fluorescein isothiocyanate (FITC)-conjugated secondary antibodies (Invitrogen) for KLK8, α-SMA, vimentin, FSP-1 at a dilution of 1:400 at 37 ºC for 1 h in the dark. Finally, nuclei were counterstained with 4'6-diamidino-2-phenylindole (DAPI) (Sigma-Aldrich). Images were observed under confocal laser scanning microscopy (LSM700; Carl Zeiss Co.,Germany) with the Z-stack technique (0.8 μm/layer), and recorded using Zeiss LSM software, version 2.3 SP1 28. The investigator performing immunofluorescence analysis was blinded to group allocation. For quantification, five high-power fields were analyzed in heart tissue sections taken from each animal. We then determined the percentages of KLK8^+^/CD31^+^ cells in total CD31^+^ cells, and the percentages of CD31^+^/α-SMA^+^, CD31^+^/FSP-1^+^ and CD31^+^/vimentin^+^ cells in total α-SMA^+^, FSP-1^+^ and vimentin^+^ cells, respectively.

HCAECs were fixed with methanol for 10 min at room temperature and incubated with PBS containing 10% goat serum, 0.3 M glycine, 1% BSA and 0.1% tween for 1 h at room temperature to permeabilise the cells and block non-specific protein-protein interactions. Cells were then incubated with primary antibodies against VE-cadherin (Santa Cruz, sc-6458) and plakoglobin (Santa Cruz, sc-7900) at a dilution of 1:100 overnight at 4 °C. After washes, cells were incubated with FITC-conjugated goat anti-goat IgG for VE-cadherin, and Alexa Fluor 568-conjugated anti-rabbit IgG for plakoglobin at a dilution of 1:400 at 37 ºC for 1 h in the dark. Finally, nuclei were counterstained with DAPI. Images were observed and photos were taken under a confocal microscope.

Primary neonatal rat cardiac fibroblasts were fixed with methanol for 5 min, permeabilized with 0.1% Triton X-100 for 5 min and then blocked with 1% BSA/10% normal goat serum/0.3 M glycine in 0.1% PBS-Tween for 1 h. The cells were then incubated with primary antibodies against Ki67 (Abcam, ab16667) at a dilution of 1:200 overnight at 4 °C, followed by a further incubation at room temperature for 1 h with Alexa Fluor 568-conjugated anti-rabbit IgG (Invitrogen, Carlsbad, CA) at a dilution of 1:400 at 37 ºC for 1 h in the dark. Finally, nuclei were counterstained with DAPI.

### Transwell migration assay

In brief, primary neonatal rat cardiac fibroblasts were infected with Ad-control or Ad-KLK8 at a multiplicity of infection (MOI) of 10 for 72 h. Then, 1×10^5^ Ad-control or Ad-KLK8-treated cardiac fibroblasts resuspended with free fetal bovine serum (FBS) medium were added to the upper compartment of migration chambers (BD Biosciences). The bottom chamber was filled with 500 μL cell culture medium with 10% FBS as an attractant. 48 h later, cells were fixed and stained with crystal violet and the migrating cells of each sample were counted in ten randomly selected fields 101.

### Statistical analysis

Statistical analyses were performed using SPSS 20 (SPSS Inc., Chicago, USA). All data are expressed as mean ± SEM. Normal distribution was assessed by Shapiro-Wilk test. Statistical significance was determined according to sample distribution and homogeneity of variance. Statistical comparisons between two groups were determined by two-tailed Student's t test. One-way or two-way ANOVA with Bonferroni's post hoc test was performed for comparisons among multiple groups. P < 0.05 was considered statistically significant.

## Supplementary Material

Supplementary figures and tables.

## Figures and Tables

**Table 1:** Blood Pressure, Heart Rate, and Blood Glucose of Streptozotocin-Induced diabetic mice

	SBP (mmHg)	DBP (mmHg)	MBP (mmHg)	HR (bpm)	Blood Glucose (mg/dL)
**Control 12 w**					
KLK8^+/+^	110.00 ± 2.86	82.61 ± 2.24	91.12 ± 2.89	530 ± 10	111 ± 2
KLK8^-/-^	108.14 ± 2.94	82.19 ± 2.19	88.67 ± 2.73	550 ± 8	113 ± 4
**Diabetes 12 w**					
KLK8^+/+^	113.27 ± 4.51	86.69 ± 3.78	97.23 ± 3.56	538 ± 10	557 ± 16****
KLK8^-/-^	111.23 ± 2.65	89.02 ± 1.69	98.58 ± 2.02	533 ± 12	537 ± 17^^^^
**Control 24 w**					
KLK8^+/+^	111.37 ± 1.39	81.20 ± 2.55	90.71 ± 2.36	577 ± 7	116 ± 5
KLK8^-/-^	113.81 ± 1.43	83.02 ± 2.28	93.57 ± 2.24	566 ± 8	114 ± 5
**Diabetes 24 w**					
KLK8^+/+^	146.69 ± 2.31**^####^**	116.37 ± 2.65**^####^**	129.22 ± 2.76**^####^**	466 ± 4**^####^**	543 ± 15**^####^**
KLK8^-/-^	126.93 ± 2.09**^$$$$^**	93.27 ± 2.17**^$$$$^**	103.87 ± 1.92**^$$$$^**	509 ± 4**^$$^**	549 ± 19

SBP indicates systolic blood pressure; DBP, diastolic blood pressure; MBP, mean blood pressure; HR, heart rate; and Blood glucose. Data are expressed as mean ± SEM (n = 7~8). ****p < 0.0001 versus KLK8^+/+^ control obtained after 12 weeks; ^^^^*p* < 0.0001 versus KLK8^-/-^ control obtained after 12 weeks; ####*p* < 0.0001 versus KLK8^+/+^ control obtained after 24 weeks; $$*p* < 0.01, $$$$*p* < 0.0001 versus KLK8^+/+^ after 24 weeks of diabetes.

**Table 2:** Echocardiographic Parameters of Streptozotocin-induced Diabetic Mice

	IVS, mm	LVEDD, mm	LVESD, mm	LVPWd, mm	FS,%	EF,%
**Control 12 w**						
KLK8^+/+^	0.73 ± 0.05	2.12 ± 0.09	1.13 ± 0.09	0.93 ± 0.06	50.73 ± 2.63	68.55 ± 2.57
KLK8^-/-^	0.81 ± 0.04	2.11 ± 0.08	1.15±0.06	1.05 ± 0.07	49.22 ± 2.26	68.98 ± 3.79
**Diabetes 12 w**						
KLK8^+/+^	0.77 ± 0.04	2.16 ± 0.11	1.10 ± 0.06	1.05 ± 0.06	48.20 ± 1.34	66.61 ± 2.27
KLK8^-/-^	0.75 ± 0.03	2.16 ± 0.08	1.19 ± 0.08	1.01 ± 0.09	51.37 ± 3.61	69.39 ± 3.46
**Control 24 w**						
KLK8^+/+^	0.81 ± 0.03	2.26 ± 0.10	1.30 ± 0.05	1.11 ± 0.08	50.76 ± 1.90	70.05 ± 1.87
KLK8^-/-^	0.77 ± 0.04	2.30 ± 0.09	1.31 ± 0.04	1.10 ± 0.06	49.65 ± 2.41	71.34 ± 3.19
**Diabetes 24 w**						
KLK8^+/+^	0.77 ± 0.03	2.86 ± 0.09****	1.69 ± 0.04****	1.18 ± 0.04	38.88 ± 1.62**	58.31 ± 1.54**
KLK8^-/-^	0.78 ± 0.02	2.53 ± 0.06**^#^**	1.41 ± 0.03**^##^**	1.06 ± 0.05	46.39 ± 1.14**^#^**	67.99 ± 1.30**^#^**

IVS indicates interventricular septum thickness; LVEDD, left ventricular end-diastolic dimension; LVESD, left ventricular end-systolic dimension; LVPWd, left ventricular diastolic posterior wall thickness; FS, fractional shortening; and EF, ejection fraction. Data are expressed as mean ± SEM (n = 7~8). **p < 0.01, ****p < 0.0001 versus KLK8^+/+^ control obtained after 24 weeks; #*p* < 0.05, ##*p* < 0.01 versus KLK8^+/+^ after 24 weeks of diabetes.

## Abbreviations

KLK8: kallikrein-related peptidase 8
KLKs: tissue kallikrein-related peptidases
EndMT: endothelial-to-mesenchymal transition
HCAECs: human coronary artery endothelial cells
TGF-β1: transforming growth factor-β1
VE-cadherin: vascular endothelial-cadherin
ECM: extracellular matrix
STZ: streptozotocin
α-SMA: α smooth muscle actin
VWF: von Willebrand factor
FSP-1: fibroblast-specific protein-1
Ad: adenovirus
KLK^-/-^: KLK8-deficient
Co-IP: co-immunoprecipitation
HIF-1α: hypoxia inducible factor-1α
HAECs: human aortic endothelial cells
CNS: central nervous system
MMPs: matrix metalloproteinases
ADAMs: a disintegrin and metalloproteinases
TCF: T-cell factor
LEF: lymphoid enhancer factor
NG: normal glucose
HG: high glucose
Tg: transgenic
MOI: multiplicity of infection
siRNA: small interference RNA
B_1_R: kinin B_1_ receptor
B_2_R: kinin B_2_ receptor
Lv: lentivirus
IP: immunoprecipitation
WB: western blot
PCNA: proliferating cell nuclear antigen
FFA: free fatty acid
TC: total cholesterol
TG: triglyceride
LDL-C: low-density lipoprotein cholesterol
HDL-C: high-density lipoprotein cholesterol
E/A: ratio of E wave to A wave amplitude
IVRT: isovolumic relaxation time
DMSO: dimethyl sulfoxide
Sp-1: specificity protein-1
