# Supplementary material for: A novel role of kallikrein-related peptidase 8 in the pathogenesis of diabetic cardiac fibrosis
Source: Theranostics. 2021 Feb 20;11(9):4207–31. doi: 10.7150/thno.48530 (PMC7977470; doi:10.7150/thno.48530)
Supplement: Supplementary file 1 — Supplementary figures and tables. [file thnov11p4207s1.pdf]

A novel role of kallikrein-related peptidase 8 in the pathogenesis of diabetic cardiac fibrosis

Jian-Kui Du[1,2a], Qing Yu[2a], Yu-Jian Liu[3], Shu-Fang Du[2], Li-Yang Huang[2], Dan-Hong Xu[3], Xin Ni[1,2]*, Xiao-Yan Zhu[2]*

[1]National Clinical Research Center for Geriatric Disorders and National International Joint Research Center for Medical Metabolomics, Xiangya Hospital, Central South University, Changsha, Hunan, China; [2]Department of Physiology, Navy Medical University, Shanghai, China; [3]School of Kinesiology, The key Laboratory of Exercise and Health Sciences of Ministry of Education, Shanghai University of Sport, Shanghai, China

[a]These authors contributed equally to this work and should be considered as co-first authors

*Corresponding Authors: Xiao-Yan Zhu, MD, PhD, Navy Medical University, 800 Xiangyin Rd, Shanghai, 200433, China. Email: xiaoyanzhu@smmu.edu.cn; Xin Ni, MD, PhD, National International Joint Research Center for Medical Metabolomics, Xiangya Hospital, Central South University, 87 Xiangya Road, Changsha, Hunan, 410008, China. Email: xinni2018@csu.edu.cn.

Table S1 Mass spectrometry analysis of the proteins pulled down by KLK8

Hits	Protein Mass (Da)	No. of Peptide	Description
1	82467	7	Plakoglobin [Rattus norvegicus]
2	26186	4	Troponin T2, cardiac, isoform CRA_a [Rattus norvegicus]
3	38939	5	Annexin II [Rattus norvegicus]
4	51391	3	Isocitrate dehydrogenase 2 (NADP+), mitochondrial [Rattus norvegicus]
5	33196	1	Adenine nucleotide translocator [Rattus norvegicus]
6	15392	2	Lipid-binding protein [Rattus norvegicus]
7	96219	1	Receptor-type tyrosine-protein phosphatase kappa isoform X5 [Rattus norvegicus]
8	71951	1	Abcf2 protein [Rattus norvegicus]
9	47658	1	Protein tyrosine phosphatase and tensin homolog/mutated in multiple advanced cancers protein [Rattus norvegicus]
10	35842	1	Olfactory receptor Olr1096 [Rattus norvegicus]

Table S2 Primer sequences used in the study

Description	Sequence 5' to 3'	Accession NO.	Species
β-actin	sense：CCACTGCCGCATCCTCTTCC<br>antisense: CTCGTTGCCAATAGTGATGACCTG	NM_007393.5	Mouse
KLK1	sense: TGCACCTCCTGTCCAGTCTC<br>antisense: GAGCAGCATCAGGTCATTGC	NM_001320331.1	Mouse
KLK2	sense：TACTACGCCTCAGCAAGCCT<br>antisense：ATTGGGTTTAGCGCATGGGA	NM_010642.3	Mouse
KLK3	sense：AGTGCTTGCCACCGAGACAT<br>antisense：GTTCCGCTTGCACTGTGCTT	NM_008455.3	Mouse
KLK4	sense：TGGCAGCCGGATGTTAGAG<br>antisense：CTCCTCCAGCGCAGAACATA	NM_019928.2	Mouse
KLK5	sense：CCACTCCAATGACCTCATGC<br>antisense：CACAGGACCTCCGGAATCA	NM_026806.2	Mouse
KLK6	sense：CCGAATCTGCAGGTGATCTTG<br>antisense：GCACCAGATGGACATCAGCA	NM_001164696.1	Mouse
KLK7	sense：GGCAATCAGCTTCACTGTGG<br>antisense：CCATCCAGAGACGGTACATGA	NM_011872.3	Mouse
KLK8	sense：GCTCTGGTGAGCCCTGCC<br>antisense：CAGCTCCGGAAACACCTCCT	NM_008940.3	Mouse
KLK9	sense：GGCTGGCCTCTTCTACCTCA<br>antisense：GGTTGCACAGCTGGAGTCAG	NM_02860.3	Mouse
KLK10	sense：CCGCTTCTTCACCTCTCCAC<br>antisense：CAAGTGGTCATCGCCAACTC	NM_133712.2	Mouse
KLK11	sense：GCCAGGATGATTCTCCGACT<br>antisense：GAAGGACTCAGTGGCCATCC	NM_001177373.1	Mouse
KLK12	sense：GCCGTGACAAGTACGTGGTG<br>antisense：GGAGAGGTTGAGGCACTGGA	NM_027097.1	Mouse
KLK13	sense：AGGTGGCTACACCTGCCTTC<br>antisense：CTGCTGAGCTGGACTGGAGA	NM_001039042.2	Mouse
KLK14	sense：ATCTCTGTGGCCAGCTCCTG<br>antisense：TAGCCAGGCATAGCACAACG	NM_174866.3	Mouse
KLK15	sense：GTGAGCACAATCTCCGCAAG<br>antisense：TGTTGGCACAATGCAACGTA	NM_174865.2	Mouse
TGF-β1	sense：ACCGCAACAACGCCATCTATGAG	NM_011577.2	Mouse

	antisense：CTTGGTTCAGCCACTGCCGTAC		
Snail	sense：ATGCACATCCGAAGCCACACG<br>antisense：GGCACTGGTATCTCTTCACATCCG	NM_011427.3	Mouse
Slug	sense：ATCGTCGGCAGCTCCACTCC<br>antisense：AACTTCTCAGCTTCGATGGCATGG	NM_011415.3	Mouse
ZEB1	sense：CCTGGCGACTGAGCATGTTGG<br>antisense：TCCTCTTCCTCCTCCTCCTC	NM_001360981.1	Mouse
ZEB2	sense:ACTCGCAGCACATGAATCACAGG<br>antisense：TCCTTCTCGCTCTCGCCATCC	NM_001289521.1	Mouse
Twist	sense：CTCGCAAGAGACGCAGCAGTC<br>antisense：AGGACCTGGTACAGGAAGTCGATG	NM_011658.2	Mouse
β-actin	sense：TGTGTTGGCGTACAGGTCTTTG<br>antisense: GGGAAATCGTGCGTGACATTAAG	NM_001101.5	human
KLK1	sense: TTCAGCACTTTCCAGTGTGG<br>antisense: AACTCCACGACCTTCACAGC	NM_002257.4	Human
KLK2	sense：CTGCCCATTGCCTAAAGAAG<br>antisense：GAACTCCTCTGGTTCGATGC	NM_001002231.2	Human
KLK3	sense：GATGCTGTGAAGGTCATGGA<br>antisense：ACTAGGGAGCCATGGAGGAC	NM_001030047.1	Human
KLK4	sense：AACAGACCCTTGCTCGCTAA<br>antisense：AGAGTCACCGTTGCAGGAGT	NM_001302961.1	Human
KLK5	sense：GAATCTGGGCAGCAGATGTT<br>antisense：TCTGTCTCGGGTAAGCATCC	NM_001077491.2	Human
KLK6	sense：CAAGCTGCCCTCTACACCTC<br>antisense：GTGGTGTTGGCTGAGCAGT	NM_001012964.3	Human
KLK7	sense：CTCATGCTCGTGAAGCTCAA<br>antisense：GGGTACCTCTGCACACCAAC	NM_001207053.2	Human
KLK8	sense：CTTCTTCAACTGCGTGACCA<br>antisense：AGTGCACCATCACACACCAG	NM_144505.3	Human
KLK9	sense：GGACTCCTCTGTGCTCTGCT<br>antisense：GCTGAGGTCCTTGTTGAAGC	NM_012315.2	Human
KLK10	sense：ATGACCACCTGCTGCTTCTT<br>antisense：GGGCTCAGGATAGTGATGCT	NM_001077500.2	Human
KLK11	sense：AGAAGGAGGAGGGCTGTGAG<br>antisense：AGGCGTTCTCACACTTCTGG	NM_001136032.3	Human
KLK12	sense：TGAGTGTGGGCGTAACTCAC<br>antisense：GGTTGAACGCTGCTGGTTA	NM_019598.2	Human
KLK13	sense：GCCTTGTCAGGAGGTGTCTC	NM_001348177.2	Human

	antisense：CATCTGAGCGAAGTTGGATG		
KLK14	sense：CATGACACAGAGCCAAGAGG<br>antisense：AGCAGCATGAGGTCGTTGT	NM_001311182.2	Human
KLK15	sense：AGCAACTACGGACCACGTCT<br>antisense：CCCTGGGTAGCTCTTGTCAC	NM_001277081.1	Human
TGF-β1	sense：AAGTGGACATCAACGGGTTC<br>antisense：GTCCAGGCTCCAAATGTAGG	NM_000660.7	Human
Snail	sense：ATGCACATCCGAAGCCACACG<br>antisense：GGCACTGGTATCTCTTCACATCCG	NM_005985.4	Human
Slug	sense：ATGCATATTCGGACCCACAC<br>antisense：GCAGATGAGCCCTCAGATTT	NM_003068.5	Human
ZEB1	sense：GCCAGCAGTCATGATGAAAA<br>antisense：TATCACAATACGGGCAGGTG	NM_001128128.3	Human
ZEB2	sense:TCGTACTCGCAGCACATGAATCAC<br>antisense：TCCTTCTCGCTCTCGCCATCC	NM_014795.4	Human
TWIST	sense：CGACGACAGCCTGAGCAACAG<br>antisense：TCCTCGTAAGACTGCGGACTCC	NM_000474.4	Human
Ki67	sense: AGCCTGTGGAAGACCTGACTGG<br>antisense：GCTGAGCCCTGTTCTTGGATGC	NM_001271366.1	Rat
PCNA	sense:CGGCGTGAACCTACAGAGCATG<br>antisense：GCACAGGAGATCACCACAGCATC	NM_022381.3	Rat
Cyclin D1	sense:CCTGCATGTTCGTGGCCTCTAAG<br>antisense：CTGCTTGTTCTCATCCGCCTCTG	XM_032891312.1	Rat

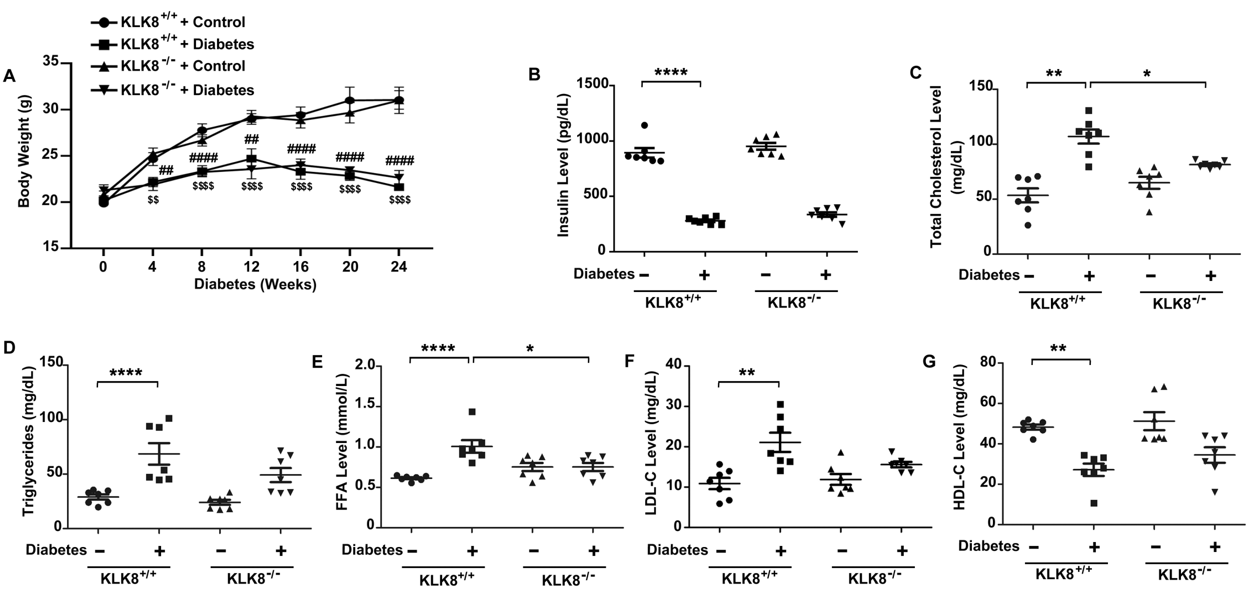

Figure S1. Effects of KLK (kallikrein-related peptidase) 8 deficiency on the body weight and phasma biochemical parameters in the streptozotocin (STZ)-induced diabetic mice. Both wild-type (KLK8$^{+/+}$) and KLK8-deficient (KLK8$^{-/-}$) mice were injected with STZ to construct diabetic mice. Body weights (A) were recorded every four weeks. Plasma levels of insulin (B), total cholesterol (TC) (C), triglyceride (TG) (D), free fatty acid (FFA) (E), low density lipoprotein cholesterol (LDL-C) (F), high density lipoprotein cholesterol (HDL-C) (G) were measured at 24 weeks after STZ injection. Data are expressed as means ± SEM (n = 7). * $p < 0.05$, ** $p < 0.01$, **** $p < 0.0001$. ## $p < 0.01$, #### $p < 0.0001$ versus KLK8$^{+/+}$ control; $$ $p < 0.01$, $$$$ $p < 0.0001$ versus KLK8$^{-/-}$ control.

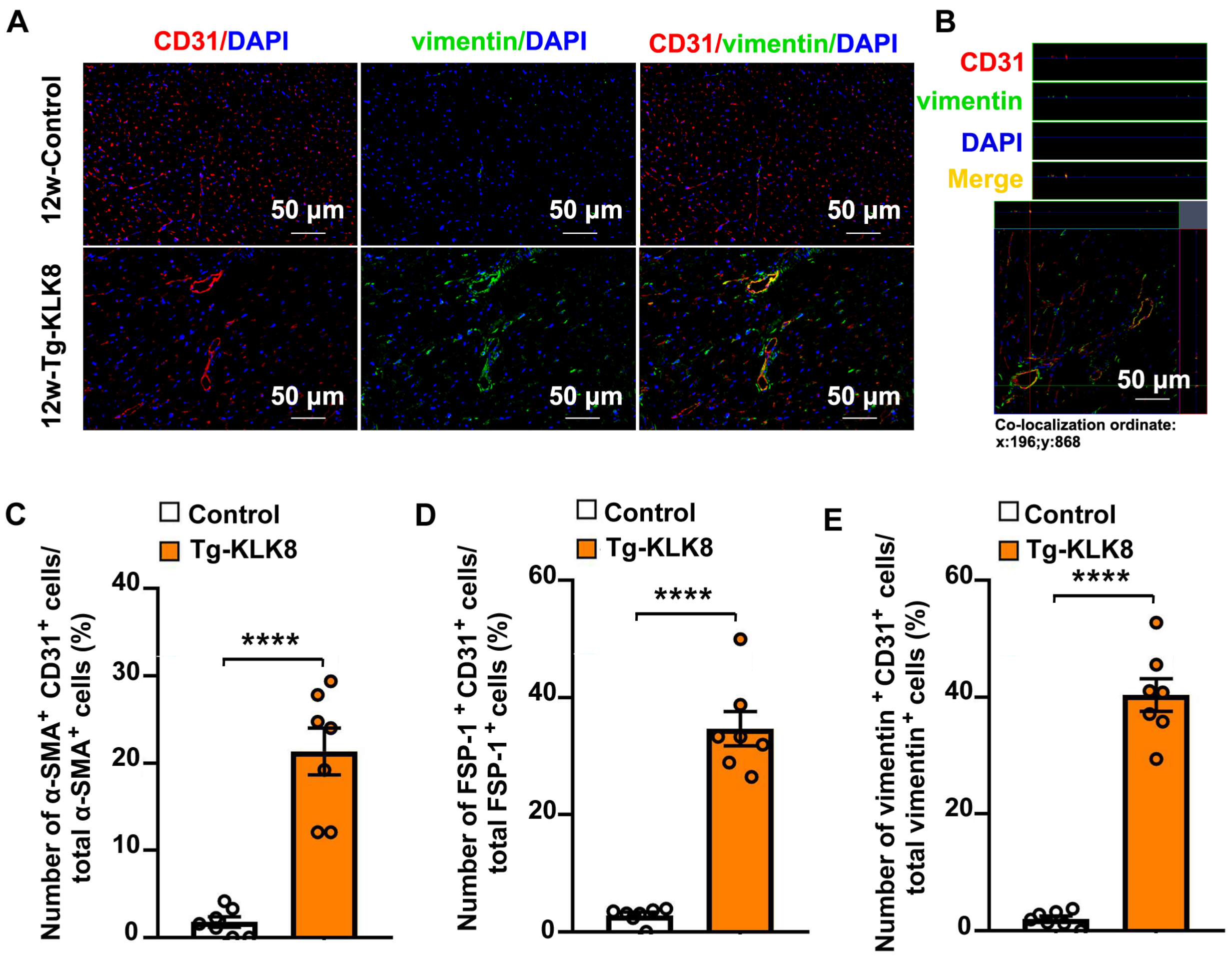

Figure S2. KLK (kallikrein-related peptidase) 8 deficiency alleviates endothelial-to-mesenchymal transition in the streptozotocin (STZ)-induced diabetic heart (Related to Figure 4). Both wild-type (KLK8$^{+/+}$) and KLK8-deficient (KLK8$^{-/-}$) mice were injected with STZ to construct diabetic mice. Heart tissues obtained at 24 weeks after STZ injection were stained with fluorophore-labeled antibodies against endothelial cell marker CD31 (red), mesenchymal markers α-SMA, FSP-1 and vimentin (green). A, Immunofluorescent staining showed decreased colocalization of mesenchymal markers vimentin (green) and the endothelial cell marker CD31 (red) in the hearts obtained from KLK8$^{-/-}$ diabetic mice as compared to KLK8$^{+/+}$ diabetic mice. Nuclei were counterstained with DAPI (blue), scale bar = 50 μm. B-D, Quantification of the percentage of CD31^{+}/α-SMA^{+} (B), CD31^{+}/FSP-1^{+} (C) and CD31^{+}/vimentin^{+} (D) cells in total α-SMA^{+}, FSP-1^{+} and vimentin^{+} cells in heart tissue sections obtained from KLK8$^{+/+}$ and KLK8$^{-/-}$ diabetic mice, respectively. Data are expressed as means ± SEM (n = 7 mice per group, 5 high-power fields per mouse). **** p< 0.0001.

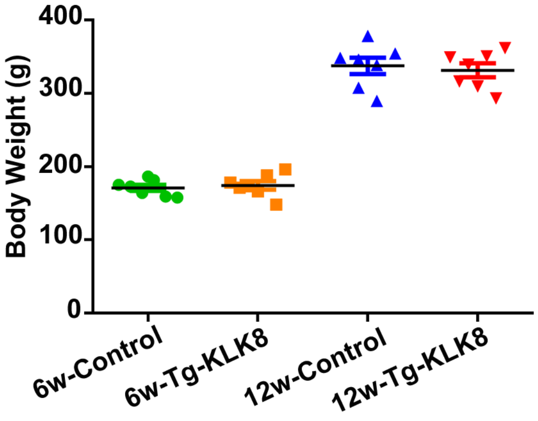

Figure S3. Body weights of 6-weeks-old and 12-weeks-old control and KLK8 transgenic (Tg-KLK8) rats. Data are expressed as means ± SEM (n = 7).

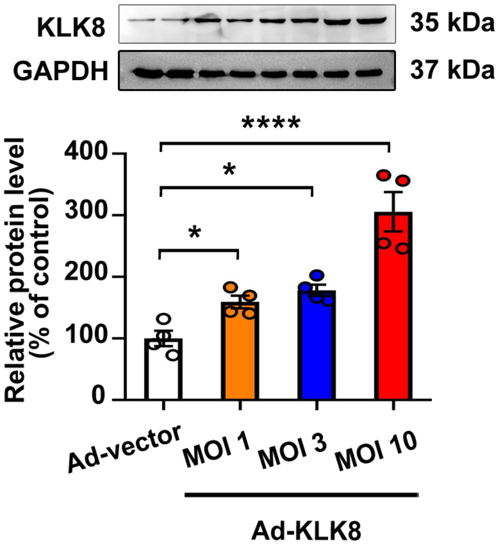

Figure S4. KLK (kallikrein-related peptidase) 8 adenovirus infection increases KLK8 expression in a dose-dependent manner in human coronary artery endothelial cells (HCAECs). HCAECs were infected with increasing doses of KLK8 adenovirus (Ad-KLK8) at a multiplicity of infection (MOI) of 1, 3, or 10 for 72 h. Immunoblots showed KLK8 expression. The representative protein bands were presented on the top of the corresponding histograms. Data are expressed as means ± SEM (n = 4). ** $p < 0.01$, **** $p < 0.0001$.

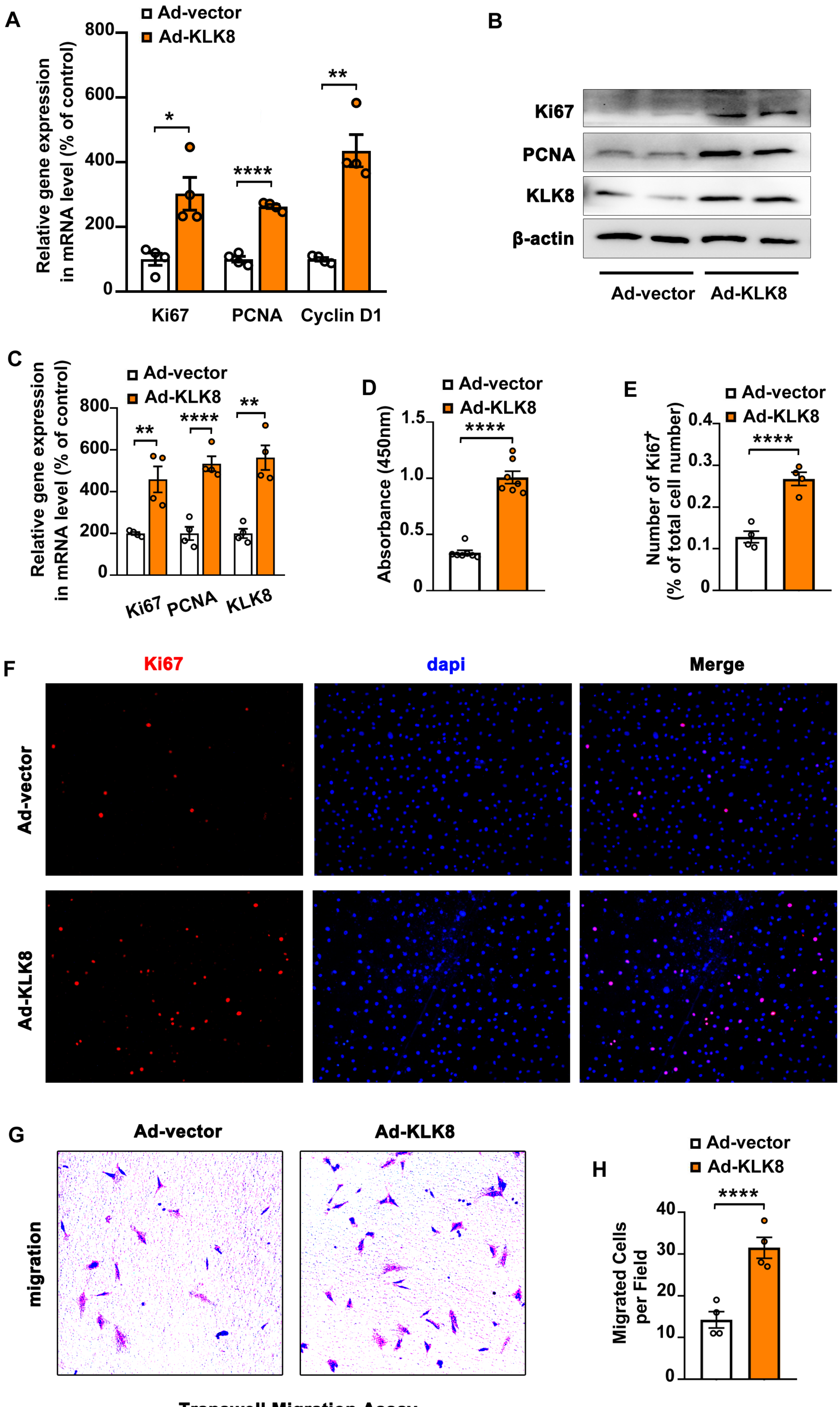
A
Ad-vector
Ad-KLK8
Relative gene expression in mRNA level (% of control)
800
600
400
200
0
*
****
**
Ki67
PCNA
Cyclin D1
B
Ki67
PCNA
KLK8
β-actin
Ad-vector
Ad-KLK8
C
Ad-vector
Ad-KLK8
Relative gene expression in mRNA level (% of control)
800
600
400
200
0
**
****
**
Ki67
PCNA
KLK8
D
Ad-vector
Ad-KLK8
Absorbance (450nm)
1.5
1.0
0.5
0
****
E
Ad-vector
Ad-KLK8
Number of Ki67+ (% of total cell number)
0.4
0.3
0.2
0.1
0
****
F
Ki67
dapi
Merge
Ad-vector
Ad-KLK8
G
Ad-vector
Ad-KLK8
migration
Transwell Migration Assay
H
Ad-vector
Ad-KLK8
Migrated Cells per Field
40
30
20
10
0
****

Figure S5. KLK (kallikrein-related peptidase) 8 overexpression promotes proliferation and migration in primary neonatal rat cardiac fibroblasts. Cardiac fibroblasts were isolated from ventricle tissues of Sprague Dawley neonatal rats (1-3 days old). Cardiac fibroblasts were infected with KLK8 adenovirus (Ad-KLK8) at a multiplicity of infection (MOI) of 10 for 72 h. A, The mRNA levels of proliferation-related genes Ki67, PCNA and cyclin D1 were increased in Ad-KLK8 treated cardiac fibroblasts. B-C, Immunoblots of Ki67, PCNA and KLK8 in cardiac fibroblasts. The representative protein bands (B) and the corresponding histograms (C) showed that Ad-KLK8 increased protein expression of Ki67, PCNA and KLK8 in cardiac fibroblasts. Cholecystokinin octapeptide (CCK8) assay (D) and the immunostaining of Ki67 (E & F) revealed that Ad-KLK8 markedly enhanced the proliferation of the cardiac fibroblasts. E, The Ki67 positive cell number (%) was shown as a ratio of the number of Ki67 positive cells to the total cell number. F, Representative Ki67-stained cardiac fibroblasts. G and H, Primary neonatal rat cardiac fibroblasts were infected with Ad-control or Ad-KLK8 at a multiplicity of infection (MOI) of 10 for 72 h. Cells were then resuspended and used for transwell migration assay as described in "Methods". Representative images of crystal violet-stained cardiac fibroblasts (G) and quantification of migrated cells per field showed that migration of Ad-KLK8-infected cardiac fibroblasts were significantly increased compared to Ad-control-infected cardiac fibroblasts. Data are expressed as means ± SEM (n = 4). * $p < 0.05$, ** $p < 0.01$, **** $p < 0.0001$.

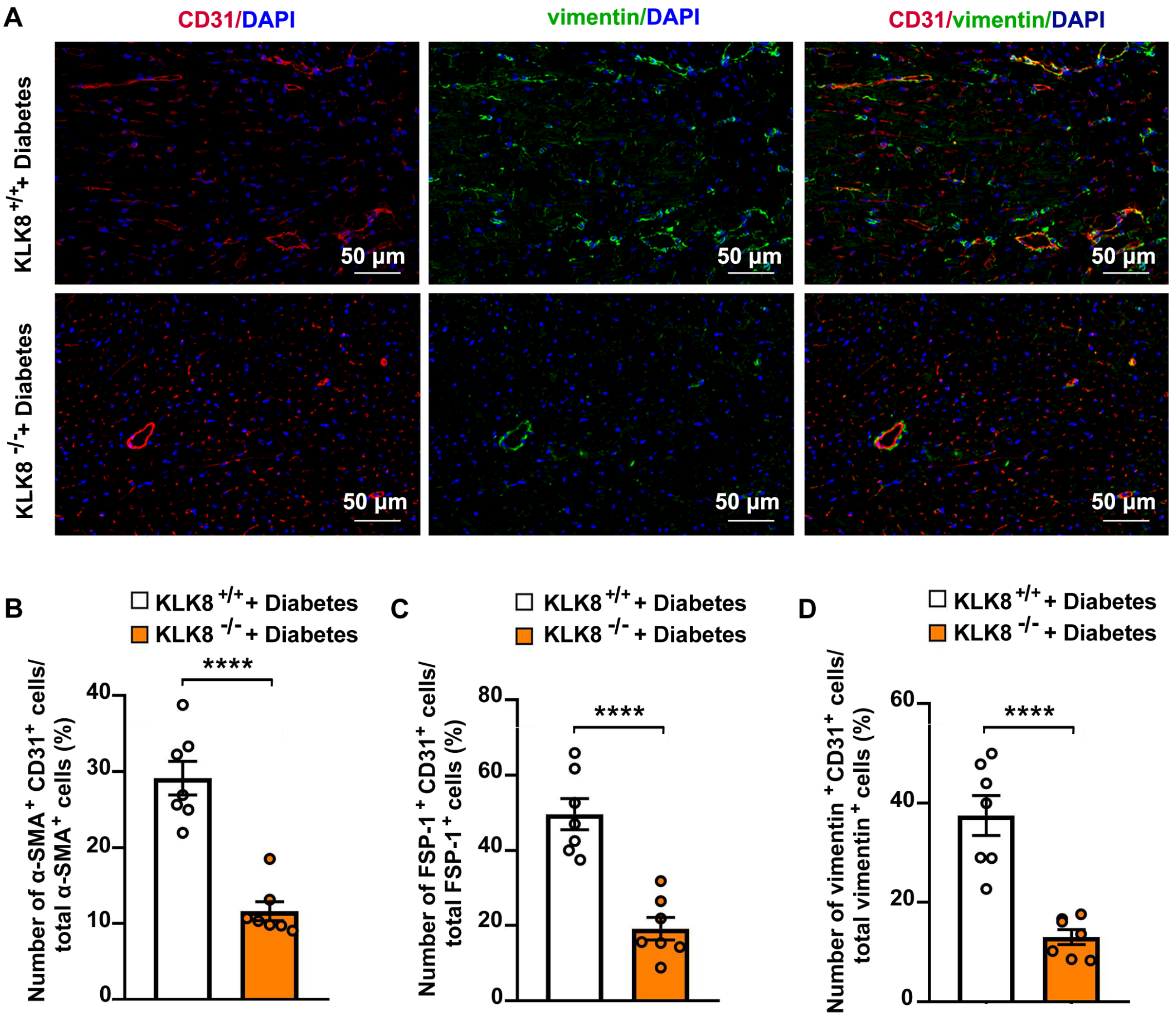

Figure S6. KLK (kallikrein-related peptidase) 8 overexpression leads to endothelial-to-mesenchymal transition in heart (Related to Figure 6). Heart tissue sections obtained from 12-weeks-old control and KLK8 transgenic (Tg-KLK8) rats were stained with fluorophore-labeled antibodies against endothelial cell marker CD31 (red), mesenchymal markers α-SMA, FSP-1 and vimentin (green). A, Immunofluorescent staining showed increased colocalization of mesenchymal markers vimentin (green) and the endothelial cell marker CD31 (red) in the hearts obtained from Tg-KLK8 rats as compared to control rats. Nuclei were counterstained with DAPI (blue), scale bar = 50 μm. B, Representative z-stack image analysis showed specific overlay of double immunostaining. $CD31^+$/$vimentin^+$ cells in specific ordinate were analyzed in z-stack with optimal interval range of 0.8 μm. Nuclei were counterstained with DAPI (blue), scale bar = 50 μm. C-E, Quantification of the percentage of $CD31^+$/α-SMA^+(C), $CD31^+$/FSP-1^+ (D) and $CD31^+$/$vimentin^+$ (E) cells in total α-SMA^+, FSP-1^+ and $vimentin^+$ cells in heart tissue sections

obtained from control and Tg-KLK8 rats, respectively. Data are expressed as means ± SEM (n = 7 rats per group, 5 high-power fields per rat). **** $p < 0.0001$.

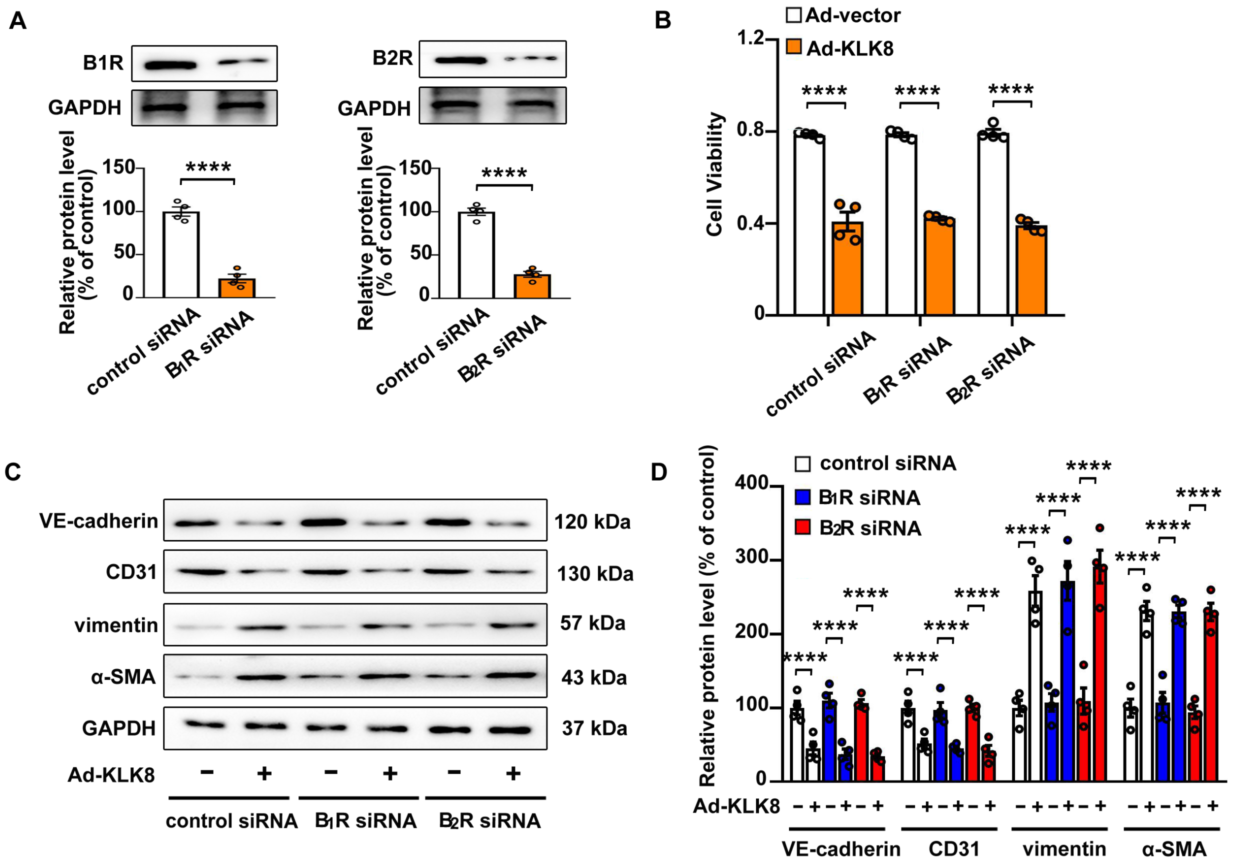

Figure S7. KLK (kallikrein-related peptidase) 8-induced endothelial cell injury and endothelial-to-mesenchymal transition is not dependent on kinin B_1R and B_2R. A, Immunoblots of kinin B_1R and B_2R in human coronary artery endothelial cells (HCAECs) transfected with siRNA targeting kinin B_1R or B_2R. B, MTT assay showed that knockdown of B_1R or B_2R could not reverse KLK8 adenovirus (Ad-KLK8)-induced cell injury in HCAECs. C&D, Immunoblots of the endothelial markers (VE-cadherin, CD31) and mesenchymal markers (vimentin, α-SMA). The representative protein bands (C) and the corresponding histograms (D) showed that knockdown of B_1R or B_2R could not reverse Ad-KLK8-induced changes in protein levels of mesenchymal and endothelial markers in HCAECs. Data are expressed as means ± SEM (n = 4). **** $p < 0.0001$.

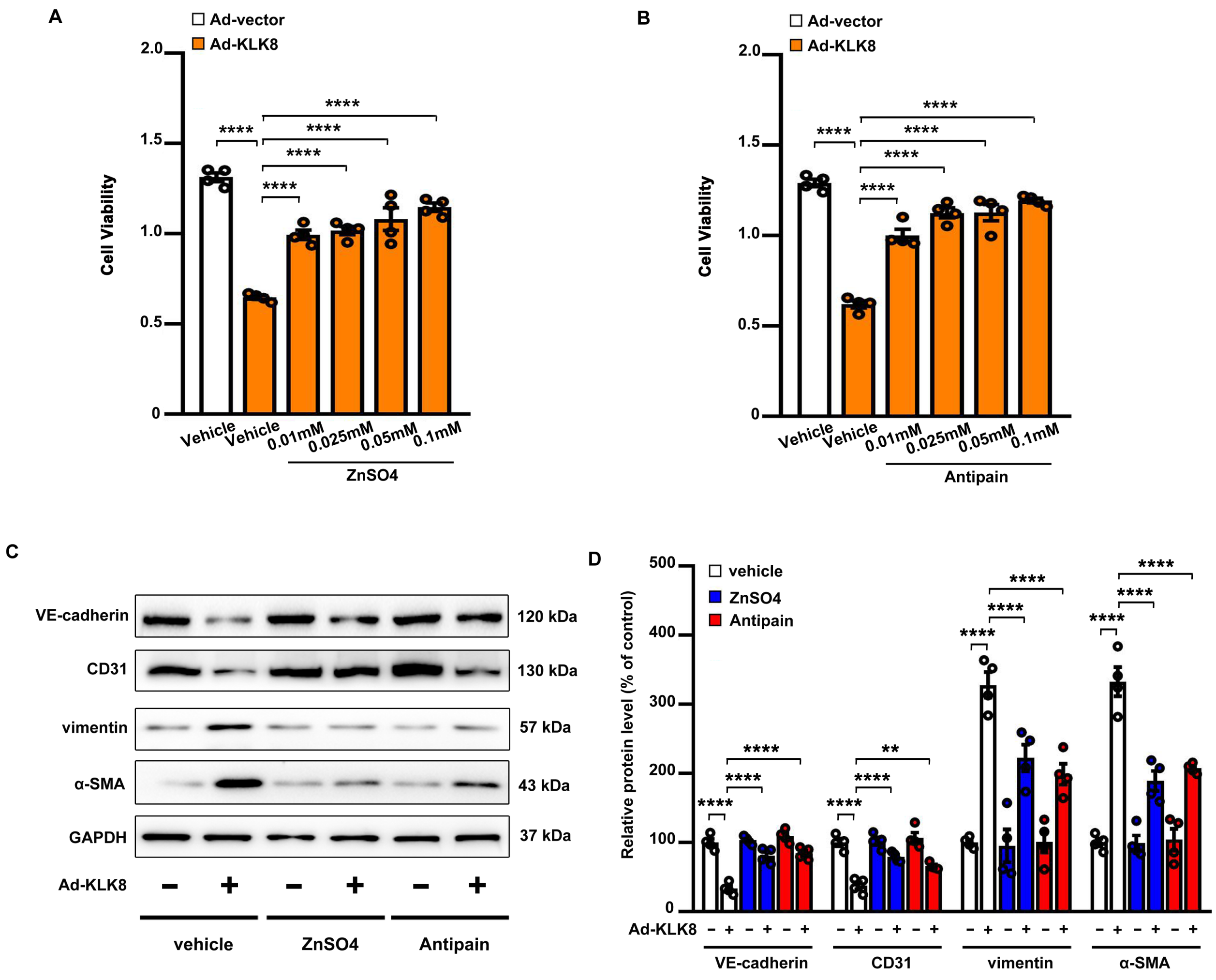

Figure S8. KLK (kallikrein-related peptidase) 8-induced endothelial cell injury and endothelial-to-mesenchymal transition is blocked by serine protease inhibitor. A and B, MTT assay showed that two serine protease inhibitors, ZnSO4 (A) and antipain (B), blocked KLK8 adenovirus (Ad-KLK8)-induced cell injury in a dose-dependent manner in human coronary artery endothelial cells (HCAECs). C&D, Immunoblots of the endothelial markers (VE-cadherin, CD31) and mesenchymal markers (vimentin, α-SMA). The representative protein bands (C) and the corresponding histograms (D) showed that two serine protease inhibitors, ZnSO4 (0.1 mM) and antipain (0.1 mM), reversed Ad-KLK8-induced changes in protein levels of mesenchymal and endothelial markers in HCAECs. Data are expressed as means ± SEM (n = 4). **** $p < 0.0001$.

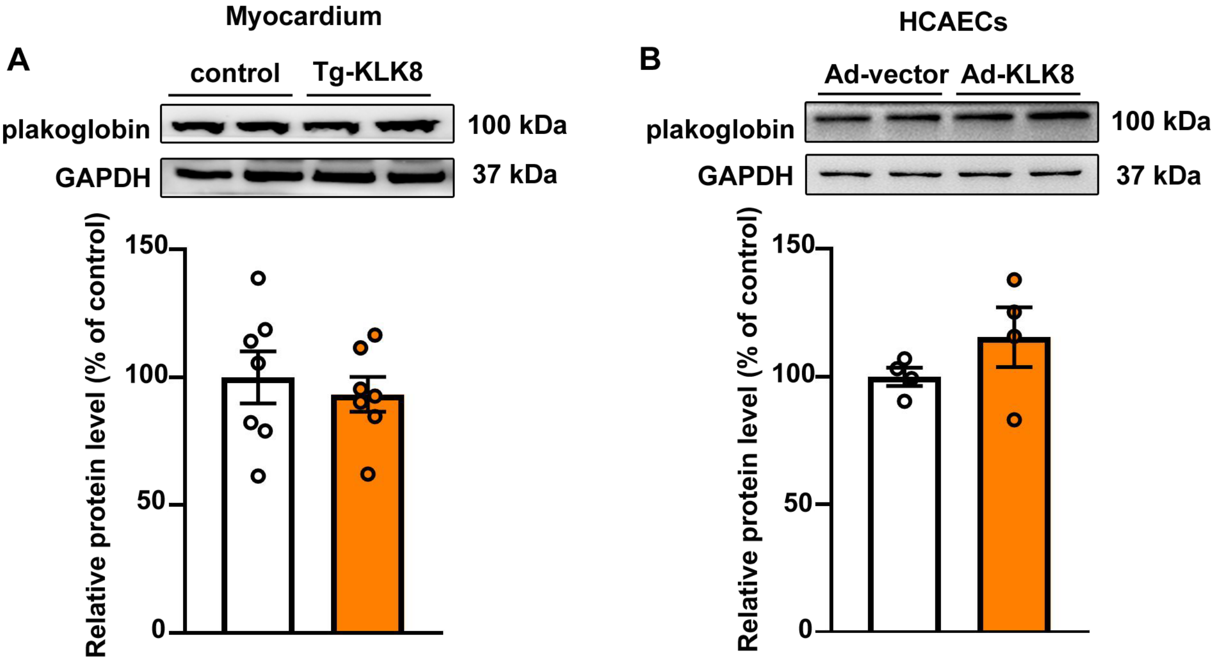

Figure S9. KLK (kallikrein-related peptidase) 8 overexpression has no significant impact on the expression of plakoglobin. Immunoblots of plakoglobin in the hearts obtained from 12-weeks-old control and Tg-KLK8 rats (A, n = 7) and human coronary artery endothelial cells (HCAECs) infected with ad-vector and KLK8 adenovirus (Ad-KLK8) for 72 h (B, n = 4). The representative protein bands were presented on the top of the corresponding histograms. Data are expressed as means ± SEM.

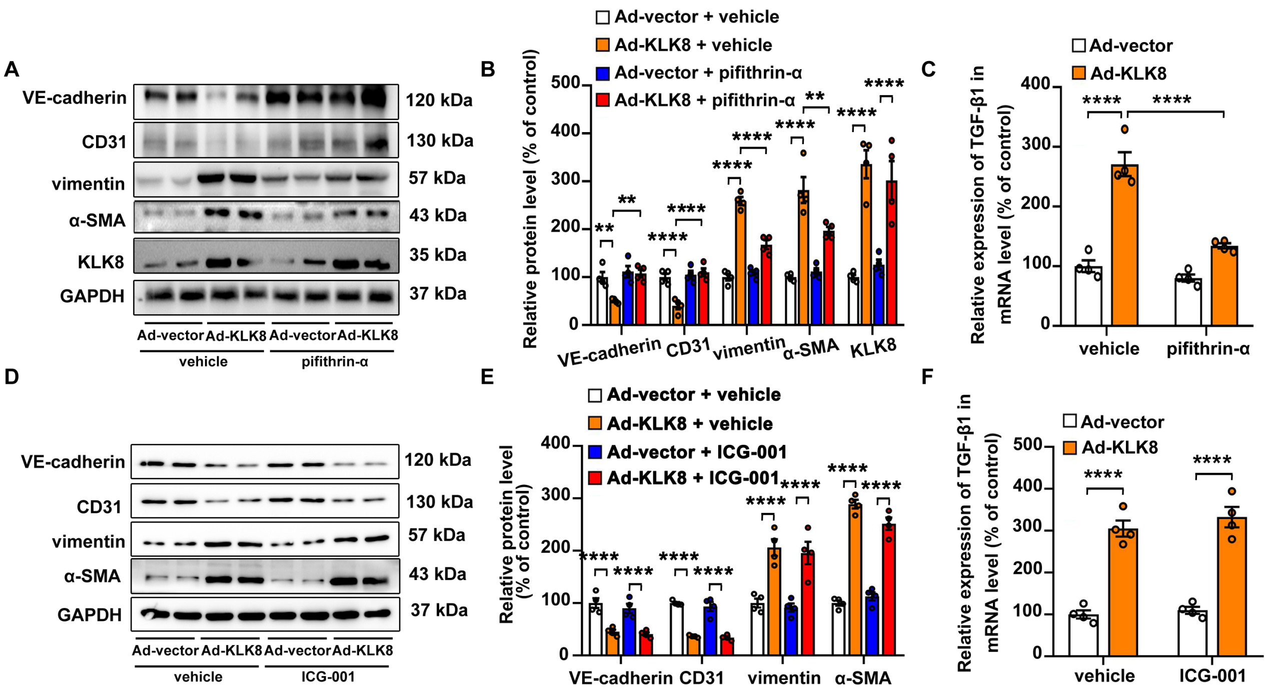

Figure S10. The effect of Pifithrin-α and ICG-001 on KLK (kallikrein-related peptidase) 8-induced EndMT and TGF-β1 mRNA expression in human coronary artery endothelial cells (HCAECs). A&B, Immunoblots of the endothelial markers (VE-cadherin, CD31) and mesenchymal markers (vimentin, α-SMA). The representative protein bands (A) and the corresponding histograms (B) showed that pifithrin-α (20 μM), an inhibitor of p53, could reverse KLK8 adenovirus (Ad-KLK8)-induced changes in protein levels of mesenchymal and endothelial markers in HCAECs. C, The KLK8 overexpression-induced mRNA expression of TGF-β1 was reduced by pifithrin-α (20 μM) in HCAECs. D&E, Immunoblots of VE-cadherin, CD31, vimentin and α-SMA. The representative protein bands (D) and the corresponding histograms (E) showed that ICG-001 (10 μM), an inhibitor of β-catenin/TCF-mediated transcription, had no effect on KLK8 adenovirus (Ad-KLK8)-induced changes in protein levels of mesenchymal and endothelial markers in HCAECs. F, ICG-001 (10 μM) could not block Ad-KLK8-induced TGF-β1 mRNA expression in HCAECs. Data are expressed as means ± SEM (n = 4). ** $p < 0.01$, *** $p < 0.001$, **** $p < 0.0001$.

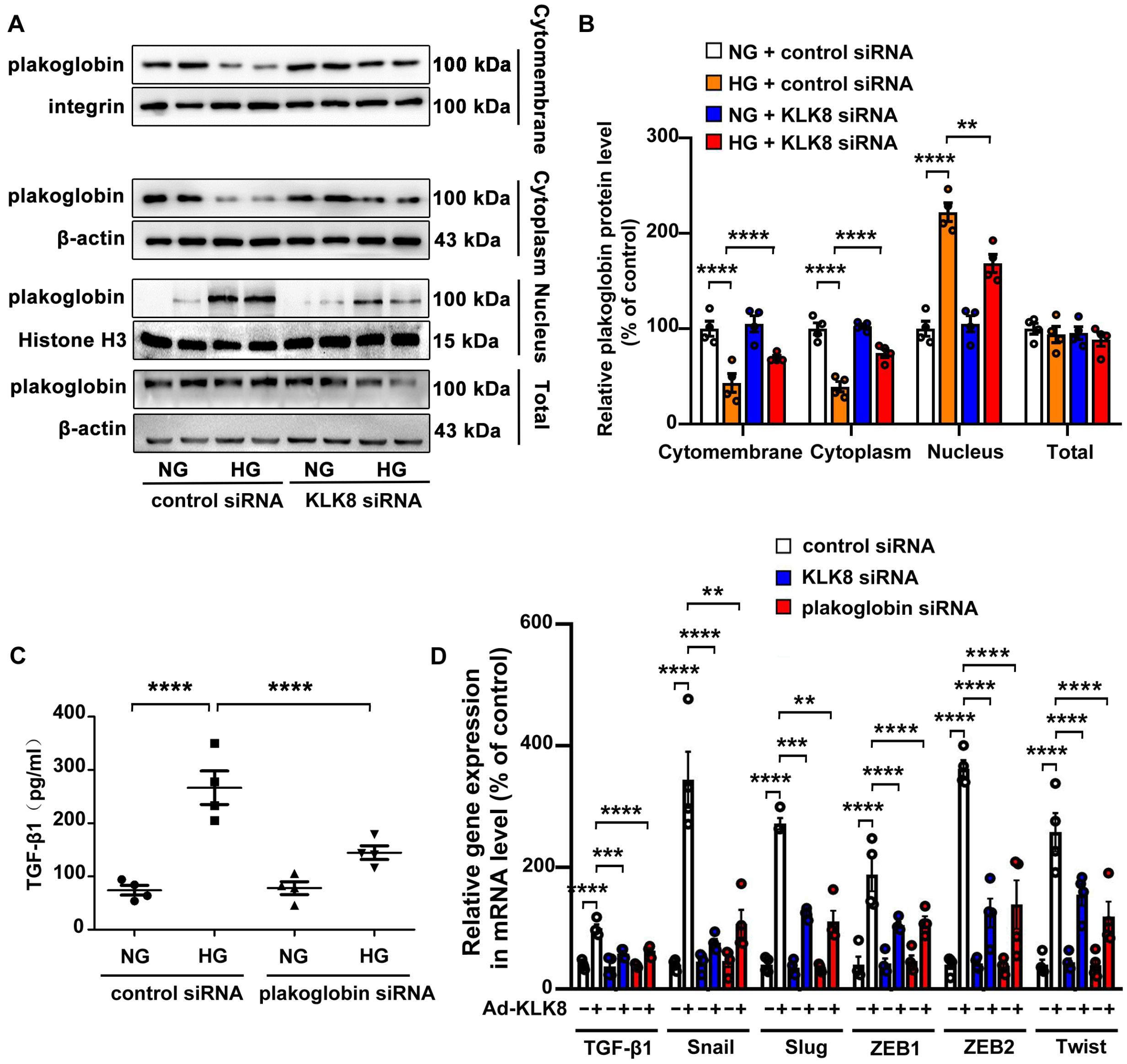

Figure S11. High glucose promotes plakoglobin nuclear translocation, meanwhile increases the expression of TGF-β1 and its pro-EndMT target genes in a KLK (kallikrein-related peptidase) 8- and plakoglobin-dependent manner. A&B, Immunoblots of cellular fractionations of plasma membrane, cytosol and nucleus for plakoglobin. The representative protein bands (A) and the corresponding histograms (B) showed that high glucose (HG, 25 mM) treatment for 5 days resulted in significant loss of membrane and cytosol plakoglobin, whereas caused profound nuclear translocation of plakoglobin in human coronary artery endothelial cells (HCAECs). KLK8 knockdown reduced nuclear whereas increased membrane and cytosol plakoglobin levels in HG-treated HCAECs. C, High glucose (HG, 25 mM) treatment for 5 days led to an increase of TGF-β1 release in the supernatant of HCAECs, which was reduced by plakoglobin knockdown. D, High glucose (HG, 25 mM) treatment for 5 days led to increases of mRNA levels of the pro-EndMT target genes of TGF-β1/Smad pathway (Snail, Slug, Zeb1, Zeb2 and Twist) in HCAECs, which was

reduced by either KLK8 or plakoglobin knockdown. Data are expressed as means ± SEM ($n = 4$). ** $p < 0.01$, *** $p < 0.001$, **** $p < 0.0001$.
